# Synthesis, Characterization, and Properties of High-Energy Fillers Derived from Nitroisobutylglycerol

**DOI:** 10.3390/ijms24108541

**Published:** 2023-05-10

**Authors:** Sergey A. Rzhevskiy, Lidiya I. Minaeva, Maxim A. Topchiy, Igor N. Melnikov, Vitaly G. Kiselev, Alla N. Pivkina, Igor V. Fomenkov, Andrey F. Asachenko

**Affiliations:** 1 A.V. Topchiev Institute of Petrochemical Synthesis, Russian Academy of Sciences, Leninsky Prospect 29, 119991 Moscow, Russia; rs89@ips.ac.ru (S.A.R.); minaeva.lidiya@gmail.com (L.I.M.); maxtopchiy@ips.ac.ru (M.A.T.); 2 Semenov Federal Research Center for Chemical Physics RAS, 4 Kosygina Str., 119991 Moscow, Russia; igor.n.melnikov@yandex.ru (I.N.M.); alla_pivkina@mail.ru (A.N.P.); 3 Institute of Chemical Kinetics and Combustion SB RAS, 3 Institutskaya Str., 630090 Novosibirsk, Russia; vitaly.kiselev@kinetics.nsc.ru; 4 Zelinsky Institute of Organic Chemistry RAS, 47 Leninsky Ave., 119991 Moscow, Russia; ootx@ineos.ac.ru

**Keywords:** energetic compounds, high-energy additives, nitroisobutylglycerol, comprehensive study, physiochemical and energetic properties

## Abstract

Herein we report a comprehensive laboratory synthesis of a series of energetic azidonitrate derivatives (ANDP, SMX, AMDNNM, NIBTN, NPN, 2-nitro-1,3-dinitro-oxypropane) starting from the readily available nitroisobutylglycerol. This simple protocol allows obtaining the high-energy additives from the available precursor in yields higher than those reported using safe and simple operations not presented in previous works. A detailed characterization of the physical, chemical, and energetic properties including impact sensitivity and thermal behavior of these species was performed for the systematic evaluation and comparison of the corresponding class of energetic compounds.

## 1. Introduction

High-energy fillers and additives are indispensable components of energetic compositions necessary to render them desirable physico-chemical, mechanical, and energy properties [1], e.g., the addition of fillers reduces the melt viscosity, elastic modulus, and glass transition temperature (T_g_) of the compositions [2]. In addition, these additives improve the energetics and oxygen balance of a mixture and affect the combustion temperature and burning rate [3]. High-energy binders can be especially useful in the production of cast-cured composites by softening and increasing the flexibility of polymer matrices, as well as improving tensile strength, elongation, toughness, and glass transition temperature [4]. Among them, the compounds with high thermal stability and low impact sensitivity are of particular interest [5,6]. It should also be noted that particularly low-molecular-weight fillers are especially promising for applications due to their simple affordable synthesis resulting in an excellent price/performance ratio [7,8].

The greatest efficiency can be achieved using the molecules containing high-energy moieties that are structurally similar to the main components of fuels, e.g., nitro (—C—NO_2_), nitramino (—N—NO_2_), difluoroamino (—NF_2_), azido (—N_3_), and nitrate ester (—ONO_2_) functional groups [9].

**Figure 1:**
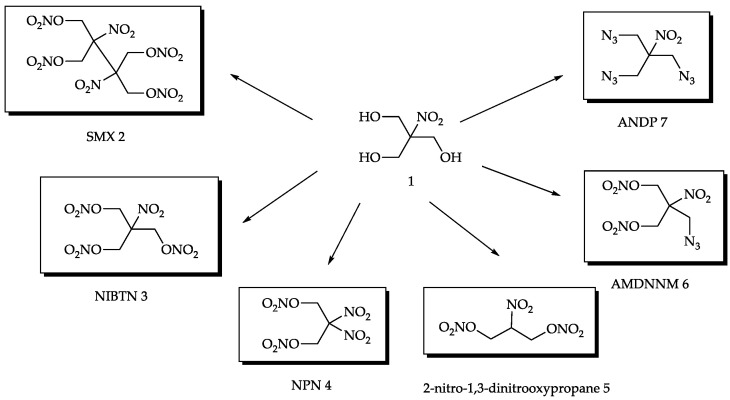
High-energy compounds derived from nitroisobutylglycerol.

Among the low-molecular high-energy derivatives of the widely available nitroisobutylglycerol [3,10,11,12,13,14,15,16,17,18,19,20,21,22,23,24,25,26,27,28,29] **1**, several compounds can be identified as promising fillers and components of various widely used composites (Figure 1).

More specifically, these are 2,3-hydroxymethyl-2,3-dinitro-1,4-butanediol tetranitrate (SMX) **2** [15,16,17], nitroisobutametriol trinitrate (NIBTN) **3** [18,19,20,21], 2,2-dinitro-1,3-bis-nitrooxy-Propane (NPN) **4** [3,22,23], 2-nitro-1,3-dinitro-oxypropane **5** [24], azidomethyl-dinitroxydimethyl-nitromethane (AMDNNM) **6** [25,26], and tris(azidomethyl)nitromethane (ANDP) **7** [27,28,29]. All of them can easily be obtained in a few simple synthetic steps from nitroisobutylglycerol **1** using cheap reagents and appear to be promising low-molecular high-energy additives according to their properties.

Usually, in a comprehensive study of high-energy additives, the following parameters should be determined [30,31]: (1) Physico-chemical properties, i.e., density, nitrogen content, oxygen balance, and melting point; (2) safety evaluation: Decomposition temperature, impact, and friction sensitivities; (3) energetic performance: Enthalpy of formation, explosion temperature, detonation pressure, and velocity. We found that these data are missing or require clarification for a number of compounds under study. For example, the heat of explosion is not available for SMX [15,16,17], and in the case of ANDP, the melting temperature and sensitivities toward mechanical stimuli are missing [27,28,29]. Apart from this, there is no information on the melting point of AMDNNM [25,26]. NPN requires the clarification of a melting point, enthalpy of formation, and detonation parameters [3,22,23]. While NIBTN has been fully characterized [18,19,20,21], the data on sensitivity and detonation parameters for 2-nitro-1,3-dinitro-oxypropane are not available at all [24]. In addition, the solid-state formation enthalpies of the compounds studied can be refined using a recently proposed combined experimental and theoretical approach [32].

All compounds **2**–**7** contain common explosophoric groups in their structure and are used as energetic additives in various compositions [3,15,18,26]. Thus, in the present contribution, we attempted to describe and systematize their synthetic procedures and determine a comprehensive set of their safety and performance parameters.

## 2. Results and Discussion

### 2.1. Synthesis

**Figure 2:**
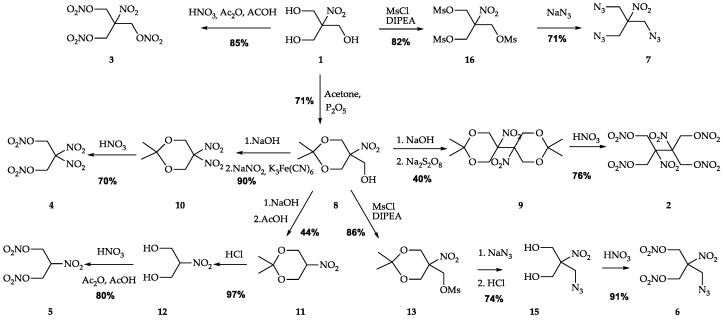
Synthesis of high-energy compounds from nitroisobutylglycerol discussed in the present work.

An overview of the syntheses is given in Figure 2. All energetic derivatives were synthesized starting from nitroisobutylglycerol **1** obtained directly from nitromethane using a well-known procedure. SMX **2** [15] and 2-nitro-1,3-dinitrooxypropane **5** [24,33] were synthesized by known methods. In turn, the synthetic procedures for NIBTN **3**, AMDNNM **6**, ANDP **7**, and NPN **4** were improved to obtain optimal yields and better working conditions.

The mixtures of concentrated nitric and sulfuric acids and oleum are commonly used in NIBTN **3** synthesis [18]. In this work, we used a mixture of nitric acid, acetic acid, and acetic anhydride as a nitrating agent. Ethyl ether was used for product extraction to avoid the formation of persistent emulsions when washing (which is often observed in methods mentioned before [18]). Flash chromatography on silica gel was used for further purification from byproducts and residual water. The traces of acetic acid in the product can be easily detected by ^1^H NMR and completely removed in vacuo. This method is more expensive, but it is reliable and convenient for obtaining a small number of pure samples in the laboratory.

For the first time, we describe a complete three-stage synthesis of NPN **4** from **1** with the 45% overall yield of the target compound. In the first step, 2,2-dimethyl-5-hydroxymethyl-5-nitro-1,3-dioxane **8** was obtained from **1** using phosphorus pentoxide [34], then 2,2-dimethyl-5,5-dinitro-1,3-dioxane **10** was prepared from **8** by adding NaNO_2_ and K_3_Fe(CN)_6_ under alkaline conditions [35]. In the third stage, **4** was isolated after treating **10** with 90% nitric acid.

The previously reported methods of **4** preparation included the treatment of **1** with concentrated nitric and sulfuric acids [22] or 100% nitric acid [36]. In our work, we used more accessible 90% nitric acid for treatment of 2,2-dimethyl-5,5-dinitro-1,3-dioxane **10**. Furthermore, the conversion of nitromethane into 2,2-dinitropropanol was described in two steps: making potassium nitromethane from undesirable chloronitromethane in the first stage and mixing it with an aqueous solution of formaldehyde [23] in the second stage with an overall 16.5% yield, which is much lower than the one reported here. AMDNNM **6** is also made from nitroisobutylglycerol **1** requiring five steps with an overall yield of 41%. Regarding the previous work on this compound [26], we increased the yield of AMDNMM from 57% to 62% (starting from (2,2-dimethyl-5-nitro-1,3-dioxan-5-yl)methanol **8** in comparison). The increase was achieved by using an additional purification stage of 2-(azidomethyl)-2-nitropropane-1,3-diol **15**. The azide derivative ANDP **7** is obtained from compound **1** in two steps using the nucleophilic substitution reaction to insert three azido groups into the trimesylate derivative of nitroisobutylglycerol. Unfortunately, there was no information on the yield of ANDP **7** in the literature [27,29]. However, it is worth mentioning that the mesylate derivative yield significantly increased from 65–68% [37,38] to 82% by using the DIPEA in DCM instead of pyridine.

All synthesized compounds were fully characterized by ^1^H, ^13^C, ^15^N NMR, and IR spectroscopies; the NMR spectra of the intermediates were also obtained.

It is also worth mentioning that the whole set of compounds **2**–**7** was synthesized and characterized in order to obtain a comprehensive comparison of their important physico-chemical properties.

### 2.2. Physico-Chemical Properties

#### 2.2.1. Crystal Structure Analysis

**Figure 3:**
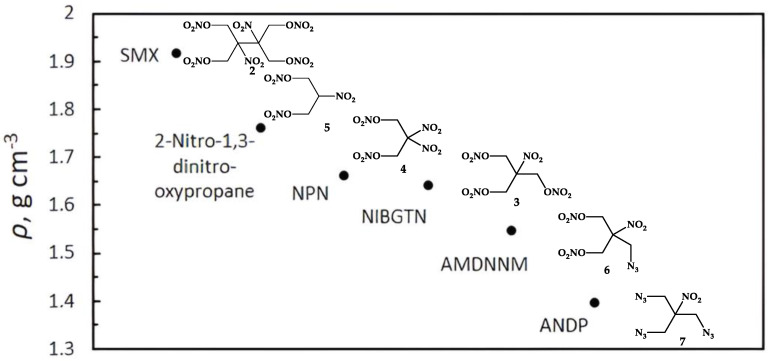
The densities of nitroisobutylglycerol derivatives.

The crystal structures of 2-nitro-1,3-dinitrooxypropane **5** [24] and SMX **2** [17] are well known in the literature. ANDP **7**, AMDNNM **6**, NIBTN **3**, and NPN **4** are liquid compounds at room temperature. The densities of **2** and **5** were calculated from the crystal structure (Figure 3).

The densities obey the order **7** < **6** < **3** < **4** in line with the increase in the oxygen content (Figure 3, ADNP 14.15% O; AMDNNM 48.1% O; NIBGTN 61.51% O; NPN 62.48% O), which is typical of the nitro compounds [39]. Compounds **5** and **2** are solid at room temperature and do not follow this trend.

#### 2.2.2. Thermal Analysis

**Figure 4:**
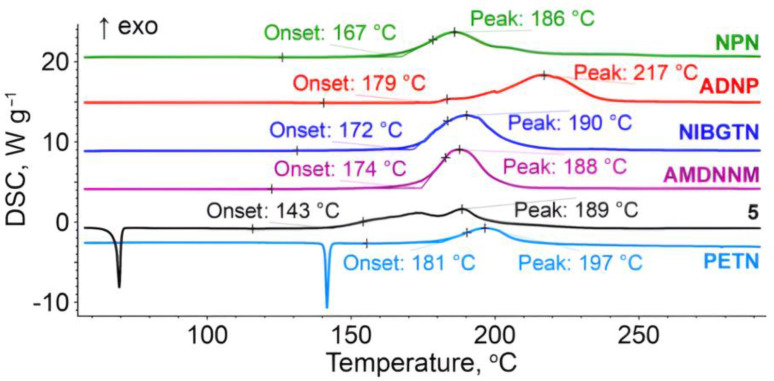
DSC curves at a heating rate of 5 K min^−1^ for nitroisobutylglycerol derivatives.

The general trends of thermal behavior of the investigated compounds are similar to the previously reported nitroesters [40,41]. More specifically, compound **4**, being a liquid at room temperature, exhibits the only thermal feature under linear heating, namely, the extrapolated onset exothermic decomposition lies at 167 °C (Figure 4). Other compounds containing the nitroester moieties, **6** and **3**, also undergo thermal decomposition within a temperature range of 160–210 °C. Compound **7** shows the best thermal stability among the species studied. Its thermolysis commences at 179 °C, but the main peak of heat release is at 217 °C, which is nearly 20 degrees higher than those of other nitroesters.

2-Nitro-1,3-dinitro-oxypropane **5** melts at 68 °C with a complex decomposition profile, having at least two distinguishable stages. The thermolysis of **5** starts at the onset temperature, which is the lowest among the considered species, whilst the second (main) exothermic DSC peak is observed at 189 °C. The latter value is nearly the same for most of the analyzed compounds. As a reference compound, PETN is also shown in Figure 4, which decomposes after melting, and the temperature range of thermolysis is approximately the same as for investigated compounds bearing nitroester groups. It should be mentioned that all species with nitroester moieties behave similarly in terms of the decomposition onset. This fact is also in line with the thermal properties of other energetic nitroethers (e.g., PETN). The only compound without several ONO_2_-groups in its structure—ADNP **7**—exhibits a retarded decomposition peak, but the decomposition onset is low due to the presence of azide groups.

In additional DSC tests with a liquid nitrogen cooling unit, we tried to crystallize the liquid nitro esters ANDP **7**, AMDNNM **6**, NIBTN **3**, and NPN **4**. All four compounds show vitrification behavior upon cooling with a 5 K min^−1^ rate to −150 °C (Figures S1–S4, Supporting information). In turn, once heated after cooling, ANDP **7** shows a sequence of thermally induced transformations: viz., a glass transition (T_g_ = −87 °C) followed by cold crystallization (−51 °C), and, finally, melting at T_m_ = 12 °C. All attempts to enhance the crystallization by the introduction of isothermal segments failed, and the vitrification and glass transition were the only thermal events observed for other samples. Thus, we see that the empirical rule T_g_/T_m_ = 2/3 (T in Kelvins [42]) remains valid for ANDP. In a similar way, we estimated the melting points for the compounds where melting was not registered directly by DSC and utilized these values in the reparametrized Trouton rule [32] to obtain the sublimation/evaporation enthalpies for the compounds studied. These vaporization enthalpies were complemented by high-level quantum chemical-calculated gas-phase formation enthalpies, and the obtained standard state formation enthalpies were used for the calculations of detonation performance. As seen from Table 1, the detonation parameters of the compounds studied range from the low energetic ANDP **7** with sub-TNT performance to a highly energetic SMX **2**, outperforming the benchmark powerful HMX explosive.

#### 2.2.3. Detonation Parameters

In Table 1 the physico-chemical properties and detonation parameters of all 6 compounds are presented.

The parameters from Table 1 show that the compounds exhibit good oxygen balance and high densities. The high densities and good oxygen balance of SMX **2** and 2-nitro-1,3-dinitro-oxypropane **5** render them to be more powerful with a detonation velocity of 9.2 and 8.3 km s^−1^, whereas lower-density NIBTN **3** and AMDNNM **6** still exhibit a good detonation velocity of 8.1 and 7.8 kms^−1^, which is comparable to PETN (8.3 km s^−1^) with 1.77 g·cm^−3^ density [45]. Compound **2** shows the largest calculated detonation pressure of 37 GPa and heat of explosion of 6260 J/g. Apart from this, 2-nitro-1,3-dinitro-oxypropane **5** and AMDNNM **6** reach the detonation pressure of 25 GPa and heat of explosion above 5800 J/g. It should also be mentioned that the oxygen balance is better for **2** and **3**, and **7** exhibits the worst oxygen balance of all compounds. In terms of sensitivity, all compounds are very sensitive towards impact (0.3–4 J) and friction (50–120 N) while PETN is classified as sensitive with 3.5 J IS and 50 N FS [46].

## 3. Materials and Methods

### 3.1. Materials

Caution: Work with explosive (or potentially explosive) materials generally requires protective apparel such as face shields, gloves, laboratory coats, and protective devices such as explosion shields, barriers, enclosed barricades, or even an isolated room. All compounds were characterized by ^1^H NMR and ^13^C NMR data. Chemicals and solvents were obtained from commercial sources and used without further purification. NMR spectra were obtained on a Bruker “Ascend™ 400” (400 MHz ^1^H, 101 MHz ^13^C, 40.55 MHz ^15^N). The chemical shifts are frequency-referenced relative to the residual undeuterated solvent peaks. Coupling constants *J* are given in Hertz as positive values regardless of their real individual signs. The multiplicity of the signals is indicated as ‘‘s”, ‘‘d”, “t”, or ‘‘m” for singlet, doublet, triplet, or multiplet, respectively. The abbreviation ‘‘br” is given for broadened signals. FT-IR spectra were obtained in a Bruker “Alpha-T” FTIR (KBr).

### 3.2. Synthetic Procedures

Preparation of 2-(hydroxymethyl)-2-nitropropane-1,3-diol **1**

A 1000 mL round-bottomed flask was charged with nitromethane (73.2 g, 1.2 mol), dry ethyl acetate (600 mL), and paraformaldehyde (108 g, 3.6 mol) followed by the addition of 33% aqueous potassium hydroxide (0.5 mL). The reaction mixture was heated under reflux with stirring in an oil bath at 100 °C, until all of the solids dissolved. The resulting mixture was cooled to room temperature and concentrated to 400 mL on a rotary evaporator. Following the addition of CHCl_3_ (1200 mL), the reaction mixture was reheated until all of the solids dissolved and then refluxed for 1 h to obtain a crystalline precipitate. The flask was sealed and placed in a freezer at −18 °C for 2 days. The precipitate was then filtered off, washed with CHCl_3_ (2 × 100 mL), and dried in vacuo to give 2-(hydroxymethyl)-2-nitropropane-1,3-diol (152.0 g, 84%) as a white solid.

M.p. 159 °C.

^1^H NMR (400 MHz, DMSO-*d6*) δ, ppm: 5.12 (t, *J* = 5.5 Hz, 3H), 3.76 (d, *J* = 5.5 Hz, 6H).

^13^C{^1^H} NMR (101 MHz, DMSO-*d6*) δ, ppm: 95.1, 58.5.

Preparation of trimethylol nitromethane trinitrate (NIBTN) **3**

A 90% HNO_3_ solution (5 mL) was added dropwise to a mixture of acetic acid (8 mL) and acetic anhydride (10 mL) and stirred for 20 min at 0 °C. Then a solution of 2-(hydroxymethyl)-2-nitropropane-1,3-diol (1.5 g, 0.01 mol) in acetic acid (4 mL) was added dropwise and the mixture was stirred for 2 h at 0 °C and for another 2 h at 20 °C. After that, the mixture was poured into 200 mL of ice and extracted with ether (2 × 50 mL). The organic layer was washed with water (3 × 100 mL), dried over Na_2_SO_4_, and evaporated in vacuo. The residue was purified by flash chromatography on silica gel (Et_2_O) to give trimethylol nitromethane trinitrate as a colorless oil (2.43 g, 85%).

^1^H NMR (400 MHz, CDCl_3_) δ, ppm: 4.99 (s, 6H).

^13^C{^1^H} NMR (101 MHz, CDCl_3_) δ, ppm: 85.3, 67.4.

^15^N NMR (40.5 MHz, CDCl_3_) δ, ppm: 373.9, 328.2.

IR (KBr): ν, cm^−1^: 1644, 1562, 1462, 1459, 1429, 1373, 1346, 1270, 1006, 814, 746, 721, 685, 615.

Preparation of (2,2-dimethyl-5-nitro-1,3-dioxan-5-yl)methanol **8**

To a mixture of tris-(hydroxymethyl)-nitromethane (15.1 g, 0.1 mol) and acetone (50 mL, 0.68 mol) phosphorus pentoxide (5 g, 0.035 mol) was added with stirring at 10 °C. The reaction mixture was stirred for 10 min, after which it was poured into 100 mL of a NaHCO_3_-saturated solution with ice. After the ice was completely dissolved, the product was filtered, washed with cold water, and dried in air to obtain 11.5 g (71%) of 2,2-dimethyl-5-hydroxymethyl-5-nitro-1,3-dioxane.

^1^H NMR (400 MHz, DMSO-d6) δ, ppm: 5.48 (t, *J* = 5.8 Hz, 1H), 4.35 (d, *J* = 13.1 Hz, 2H), 4.05 (d, *J* = 13.1 Hz, 2H), 3.73 (d, *J* = 5.8 Hz, 2H), 1.40 (s, 3H), 1.25 (s, 3H).

^13^C{^1^H} NMR (101 MHz, DMSO-d6) δ, ppm: 98.4, 87.5, 62.4, 61.2, 26.8, 20.1.

Preparation of 2,2,2′,2′-tetramethyl-5,5′-dinitro-5,5′-bi(1,3-dioxane) **9**

A 1000 mL round-bottomed flask, equipped with a reflux condenser and a magnetic stir bar, was charged with (2,2-dimethyl-5-nitro-1,3-dioxan-5-yl) methanol (41.37 g, 0.216 mol) and a solution of sodium hydroxide (17.31 g, 0.432 mol) in water (542 mL) at an ambient temperature. The reaction mixture was heated at 60 °C for 1 h and then cooled to 20 °C. Portions of solid sodium peroxodisulfate (103.05 g, 0.432 mol) were added and stirred at 20 °C for 20 h. Maintaining a temperature of no higher than 25 °C, a 40% aqueous sodium hydroxide solution was added to the resulting mixture to pH > 11, and the precipitated crystals were filtered off and washed with water on the filter. The solid was then dissolved in methylene chloride (200 mL), washed with water [TLC control (CH_2_Cl_2_) until any impurities had disappeared, approximately 5 times 50 mL each], dried over anhydrous sodium sulphate, and concentrated in a rotary evaporator to a volume of 30 mL.

The product was purified by chromatography on silica gel (eluent-methylene chloride) to give 2,2,2′,2′-tetramethyl-5,5′-dinitro-5,5′-bi(1,3-dioxane) (13.9 g, 40%) as a colorless fine crystalline powder.

^1^H NMR (400 MHz, CDCl_3_) δ, ppm: 4.45 (d, *J* = 13.6 Hz, 4H), 4.34 (d, *J* = 13.5 Hz, 4H) 1.42 (s, 6H), 1.36 (s, 6H).

^13^C{^1^H} NMR (101 MHz, CDCl_3_) δ, ppm: 100.8, 90.1, 60.5, 24.1, 21.9.

Preparation of 1,4-dinitrato-2,3-dinitro-2,3-bis(nitratomethyl)butane (SMX) **2** [15]

A 50 mL round-bottomed flask, equipped with a magnetic stir bar, was charged with 98% nitric acid (33 mL, 49.5 g, 0.78 mol) and cooled to 0 °C. 2,2,2′,2′- Tetramethyl-5,5′-dinitro-5,5′-bi(1,3-dioxane) (2.4 g, 7.5 mmol) was then added at 0 °C. The reaction mixture was stirred for 3 h at 0 °C, then poured into ice water (170 mL) and stirred for an additional 15 min at room temperature to crystallize the precipitated oily liquid. The yellowish crystals were filtered off, dissolved in methylene chloride (50 mL), and the organic phase was washed with a solution of NaHCO_3_ (2 × 50 mL). After drying over Na_2_SO_4_, the solvent was evaporated in vacuo. The resulting product was transferred to a filter, washed with diethyl ether (2 × 4 mL), and dried in vacuo to give 1,4-dinitrato-2,3-dinitro-2,3-bis(nitratomethyl)butane (2.41 g, 76%) as a colorless fine crystalline powder.

^1^H NMR (400 MHz, CDCl_3_) δ, ppm: 5.26 (d, *J* = 12.2 Hz, 4H), 5.09 (d, *J* = 12.2 Hz, 4H).

^13^C{^1^H} NMR (101 MHz, CDCl_3_) δ, ppm: 89.0, 66.1.

IR (KBr): ν, cm^−1^: 3045, 3028, 2982, 2927, 1658, 1583, 1491, 1465, 1450, 1390, 1371, 1334, 1287, 1156, 1099, 1056, 1022, 995, 898, 854.

^15^N NMR (40.5 MHz, CDCl_3_) δ, ppm: 325.0, 357.5.

Preparation of 2,2-dimethyl-5,5-dinitro-1,3-dioxane **10**

In a 250 mL round-bottomed flask equipped with a magnetic stir bar, 2,2-dimethyl-5-hydroxymethyl-5-nitro-1,3-dioxane (1.5 g, 0.37 mol) was dissolved in 48 mL of a 20% NaOH solution and stirred for 20 min at room temperature. Then, the solution of NaNO_2_ (21 g, 0.30 mol) in 24 mL of H_2_O was added, and the resulting homogeneous mixture was quickly poured into 250 mL of a K_3_Fe(CN)_6_ saturated solution. The reaction mixture was stirred for 4 h at room temperature and extracted with ethyl acetate (4 × 100 mL). The extract was washed with brine and water and dried over sodium sulphate. The crude product was concentrated on a rotary evaporator, and purified by preparative chromatography (SiO_2_, hexane/CH_2_Cl_2_ = 1/1). Furthermore, 11 g (90%) of 2,2-dimethyl-5,5-dinitro-1,3-dioxane was obtained.

^1^H NMR (400 MHz, CDCl_3_) δ, ppm: 4.67 (s, 2H), 1.47 (s, 3H).

^13^C{^1^H} NMR (101 MHz, CDCl_3_), δ, ppm: 101.1, 62.3, 23.1.

Preparation of 2,2-dinitropropane-1,3-diyl dinitrate (NPN) **4**

2,2-Dimethyl-5,5-dinitro-1,3-dioxane (4.12 g, 0.02 mol) was added with vigorous stirring to 60 mL of 90% nitric acid cooled to −20 °C in 100 mL round-bottomed flask, equipped with a magnetic stir bar. The reaction mixture was stirred for 3 h at a temperature not exceeding 5 °C before being poured into 300 mL of ice water. The crude product was extracted with methylene chloride (4 × 75 mL), washed with water and sodium carbonate saturated solution, dried by filtration through a small plug of silica gel, and concentrated and purified by preparative chromatography (SiO_2_, hexane/EtOAc = 10/1) to result in 3.58 g (70%) of 2,2-dinitropropane-1,3-diyl dinitrate.

^1^H NMR (400 MHz, CDCl_3_) δ, ppm: 5.41 (s, 2H).

^13^C{^1^H} NMR (101 MHz, CDCl_3_) δ, ppm: 110.5, 66.32.

^15^N NMR (40.5 MHz, CDCl_3_) δ, ppm: 324.7, 358.6.

IR (KBr): ν, cm^−1^: 3685, 3606, 3340, 3029, 2978, 2940, 2896, 2653, 2553, 2441, 2114, 1960, 1877, 1674, 1582, 1454, 1425, 1387, 1347, 1365, 1281, 1208, 1180, 1129, 1026, 940, 828, 743, 675, 629, 579, 504.

Preparation of 2,2-dimethyl-5-nitro-1,3-dioxane **11**

(2,2-Dimethyl-5-nitro-1,3-dioxan-5-yl)methanol (5.7 g, 0.03 mol) was dissolved in 52 mL of a 10% NaOH solution and stirred at 60 °C for 1 h in a 250 mL round-bottomed flask, equipped with a magnetic stir bar. After that, the reaction mixture was cooled to 5 °C and neutralized with 15% acetic acid (100 mL). The precipitate was filtered off and recrystallized from petroleum ether to give 2,2-dimethyl-5-nitro-1,3-dioxane in a 1.6 g (44%) yield.

^1^H NMR (400 MHz, CDCl_3_) δ, ppm: 4.48 (dd, *J* = 13.1, 4.1 Hz, 2H), 4.35 (p, *J* = 4.1 Hz, 1H), 4.24 (dd, *J* = 13.0, 4.0 Hz, 2H), 1.45 (s, 3H), 1.41 (s, 3H).

^13^C{^1^H} NMR (101 MHz, CDCl_3_) δ, ppm: 99.3, 77.3, 59.9, 25.5, 21.4.

Preparation of 2-nitropropane-1,3-diol **12**

In a 50 mL round-bottomed flask, 2,2-dimethyl-5-nitro-1,3-dioxane (1.35 g, 0.008 mol) was stirred with 3 mL of hydrochloric acid in 30 mL of methanol at 60 °C for 24 h, then the solvent was evaporated in vacuo to dryness and the mixture was purified by flash chromatography (DCM/methanol = 1/10, then 1/5) to give 2-nitropropane-1,3-diol in 0.95 g (97%) yield.

^1^H NMR (400 MHz, DMSO-d6) δ, ppm: 5.26 (s, 2H), 4.70–4.60 (m, 1H), 3.82–3.66 (m, 4H).

^13^C{^1^H} NMR (101 MHz, DMSO-d6) δ, ppm: δ 92.4, 60.0.

Preparation of 2-nitro-1,3-dinitrooxypropane **5** [24]

To a mixture of acetic anhydride (7 mL) and acetic acid (5 mL), 90% nitric acid (2.8 mL) was added dropwise at 0 °C and stirred for 20 min. Then, a solution of 2-nitropropane-1,3-diol (0.95 g, 0.0078 mol) in 2 mL of acetic acid was added dropwise and stirred for another 2 h at 0 °C. The reaction mixture was poured into 25 mL of ice, the precipitated crystals were filtered, washed with water on the filter, and recrystallized from CCl_4_ to obtain (1.3 g, 80%) 2-nitro-1,3-dinitrooxypropane.

^1^H NMR (400 MHz, acetone-d6) δ, ppm: 5.60–5.52 (m, 1H), 5.34–5.22 (m, 4H).

^13^C{^1^H} NMR (101 MHz, acetone-d6) δ, ppm: 81.3, 69.6.

^15^N NMR (40.5 MHz, CDCl_3_) δ, ppm: 330.4, 375.9.

IR (KBr): ν, cm^−1^: 3434, 3309, 3032, 2985, 2933, 2798, 2709, 2570, 2424, 1767, 1659, 1676, 1562, 1500, 1468, 1437, 1404, 1339, 1355, 1310, 1285, 1254, 1229, 1116, 1082, 1052, 1013, 989, 911, 837, 749, 691, 627, 646, 600, 513, 465.

Preparation of (2,2-dimethyl-5-nitro-1,3-dioxan-5-yl)methyl methanesulfonate **13**

A solution of (2,2-dimethyl-5-nitro-1,3-dioxan-5-yl)methanol (5.0 g, 0.026 mol) in dry methylene chloride (70 mL) was cooled to −10 °C and DIPEA (5 mL, 0.028 mol) was added in portions, then the mixture was stirred for 15 min at this temperature. Then, mesyl chloride (2.22 mL, 0.028 mol) was added dropwise and stirred for another hour at −5 °C to 5 °C. The reaction mixture was warmed to room temperature and stirred for another 2 h, then washed 3 times with 100 mL of water, dried over Na_2_SO_4_, and purified by flash chromatography on silica gel (DCM) to give (2,2-dimethyl-5-nitro-1,3-dioxan-5-yl)methyl methanesulfonate in 6 g (86%) yield.

^1^H NMR (400 MHz, DMSO-d6) δ, ppm: δ 5.55–5.43 (m, 1H), 4.35 (dd, *J* = 13.1, 1.4 Hz, 2H), 4.11–3.97 (m, 2H), 3.78–3.63 (m, 2H), 3.36 (d, *J* = 1.3 Hz, 3H), 1.40 (s, 3H), 1.25 (s, 3H).

^13^C{^1^H} NMR (101 MHz, DMSO-d6) δ, ppm: 98.9, 87.9, 62.8, 61.6, 27.3, 20.5.

Preparation of 5-(azidomethyl)-2,2-dimethyl-5-nitro-1,3-dioxane **14**

(2,2-Dimethyl-5-nitro-1,3-dioxan-5-yl)methyl methanesulfonate (3.0 g, 0.008 mol) was stirred for 18 h with sodium azide (1.1 g, 0.016 mol) in DMF (25 mL) at 95 °C. The reaction mixture was cooled to ambient temperature, poured into cool water, extracted with ethyl acetate (3 × 100 mL), dried over Na_2_SO_4_, and the solvent was evaporated in vacuo. The residue was purified by flash chromatography on silica gel (hexane-DCM 1/1), handled with potassium permanganate (Rf 0.67 DCM) to give 5-(azidomethyl)-2,2-dimethyl-5-nitro-1,3-dioxane (1.8 g, 76%).

^1^H NMR (400 MHz, CDCl_3_) δ, ppm: 4.36 (d, *J* = 12.6 Hz, 2H), 4.00 (d, *J* = 12.6 Hz, 2H), 3.96 (s, 2H), 1.44 (s, 3H), 1.41 (s, 3H).

^13^C{^1^H} NMR (101 MHz, CDCl_3_), δ, ppm: 84.2, 62.1, 52.8, 24.6, 22.0.

Preparation of 2-(azidomethyl)-2-nitropropane-1,3-diol **15**

5-(Azidomethyl)-2,2-dimethyl-5-nitro-1,3-dioxane (4.2 g, 0.019 mol) was stirred in a mixture of HCl (7 mL) and methanol (70 mL) at 60 °C for 17 h. Afterward, the mixture was evaporated in vacuo and the residue was purified by flash chromatography (DCM/Methanol = 20/1 then 5/1) to give 2-(azidomethyl)-2-nitropropane-1,3-diol (2.3 g, 97%).

^1^H NMR (400 MHz, DMSO-d6) δ, ppm: 5.56–5.42 (m, 2H), 3.91 (s, 2H), 3.82–3.68 (m, 4H).

^13^C{^1^H} NMR (101 MHz, DMSO-d6) δ, ppm: 93.9, 60.1, 48.6.

Preparation of 2-(azidomethyl)-2-nitropropane-1,3-diyl dinitrate (AMDNNM) **6**

A 90% HNO_3_ solution (5 mL) was added dropwise to a mixture of acetic acid (9.4 mL) and acetic anhydride (15 mL) and stirred for 20 min at 0 °C. Then a solution of 2-(azidomethyl)-2-nitropropane-1,3-diol (2.27 g, 0.013 mol) in acetic acid (4 mL) was added dropwise and the mixture was stirred for 2 h at 0 °C and for another 2 h at 20 °C. After that, the mixture was poured into 200 mL of ice and extracted with ether (2 × 150 mL). The organic layer was washed with water (3 × 100 mL) and saturated with NaHCO_3_ solution, dried over Na_2_SO_4_, and evaporated in vacuo. The residue was purified by flash chromatography on silica gel (hexane/DCM = 2/1) to give 2-(azidomethyl)-2-nitropropane-1,3-diyl dinitrate as a colorless oil (3.12 g, 91%).

^1^H NMR (400 MHz, CDCl_3_) δ, ppm: 4.94 (s, 4H), 4.01 (s, 2H).

^13^C{^1^H} NMR (101 MHz, CDCl_3_) δ, ppm: 86.5, 67.8, 50.4.

^15^N NMR (40.5 MHz, CDCl_3_) δ, ppm: 244.4, 329.2, 376.6.

IR (KBr): ν, cm^−1^: 3683, 3369, 3023, 2926, 2680, 2554, 2120, 1888, 1825, 1659, 1561, 1444, 1460, 1373, 1394, 1345, 1278, 1130, 1027, 946, 838, 750, 720, 688, 633, 550.

Preparation of 2-((methylsulfonyloxy)methyl)-2-nitropropane-1,3-diyl dimethanesulfonate **16**

DIPEA (50 mL) was added dropwise to a cooled mixture of 2-(hydroxymethyl)-2-nitropropane-1,3-diol (13 g, 0.086 mol) in DCM (250 mL) at −10 °C and stirred for 15 min at this temperature. Then MsCl (22 mL, 0.284 mol) was added dropwise for 1 h at −15 °C. The mixture was stirred for 1 h at −15 °C, then heated to 5 °C and filtered. The precipitate was washed with DCM, cold methanol, and the filtrate was evaporated. The precipitate was washed with ice water, filtered, and reprecipitated from acetone/methanol to obtain 2-((methylsulfonyloxy)methyl)-2-nitropropane-1,3-diyl dimethanesulfonate (27 g, 82%).

^1^H NMR (400 MHz, acetone-d6) δ, ppm: 4.86 (s, 1H), 3.28 (s, 2H).

^13^C{^1^H} NMR (101 MHz, acetone-d6) δ, ppm: 89.1, 65.7, 37.5.

Preparation of 1,3-diazido-2-(azidomethyl)-2-nitropropane (ANDP) **7**

2-((Methylsulfonyloxy)methyl)-2-nitropropane-1,3-diyl dimethanesulfonate (12.0 g, 0.031 mol) was stirred with sodium azide (12.1 g, 0.187 mol) in DMF (100 mL) at 95 °C overnight, then poured into ice (200 g) and extracted with EtOAc (3 × 100 mL). The organic extract was washed with water, dried over sodium sulfate, and concentrated under reduced pressure to give a residue that was chromatographed to obtain pure 1,3-diazido-2-(azidomethyl)-2-nitropropane (4,99 g, 71%).

^1^H NMR (400 MHz, CDCl_3_) δ, ppm: 3.88 (s, 6H).

^13^C{^1^H} NMR (101 MHz, CDCl_3_) δ, ppm: 89.4, 51.1.

^15^N NMR (40.5 MHz, CDCl_3_) δ, ppm: 245.15, 382.58.

IR (KBr): ν, cm^−1^: 3376, 2937, 2885, 2527, 2112, 1960, 1727, 1662, 1556, 1447, 1380, 1339, 1356, 1289, 1079, 941, 963, 918, 893, 863, 743, 661, 551, 519, 488

### 3.3. Methods

Thermal analysis runs were carried out using the DSC 204 HP (Netzsch) apparatus. Samples of 0.3–1.0 mg mass were loaded in standard aluminum crucibles and covered with pierced lids to relieve the pressure build-up by gas decomposition products. A heating rate of 5 K min^−1^ and nitrogen flow within the furnace were applied to the samples. To investigate the thermal behavior of liquid compounds, the LN_2_ cooling unit was used, and the additional step of cooling with a 5 K min^−1^ rate down to −150 °C was introduced.

Electronic structure calculations were performed using the Gaussian 09 [47], Molpro 2010 [48], and ORCA 4.0 [49] program packages. The gas-phase enthalpies of formation (at p^0^ = 1 bar and T = 298.15 K, Δ_f_H_m_^0^(g)) were calculated using the explicitly correlated W1-F12 multi-level procedure and atomization energy and isodesmic reaction approaches described in detail elsewhere [50,51,52,53]. As a general rule, the explicitly correlated F12 procedure markedly accelerates the slow basis set convergence of conventional CCSD(T) techniques [54]. Note that the W1-F12 procedure employed in the present work had been slightly modified in comparison with the originally proposed technique; namely, the B3LYP-D3BJ/def2-TZVPP-optimized geometries (with a ZPE correction factor of 0.99) were used [43,55], and the diagonal Born-Oppenheimer corrections were omitted. The multireference character of the wave functions of the reagents, intermediates, and transition states considered in the present work was estimated using the T1 diagnostic for the CCSD calculation [56]. The modest T1 values obtained in all cases (<0.025) justify the reliability of the single reference-based electron correlation procedure employed in the present study. The heats of formation at 0 K for the elements in the gas phase Δ_f_H_m_^0K^(g) [H] = 216.04 kJ mol^−1^, Δ_f_H_m_^0K^(g) [C] = 711.19 kJ mol^−1^, Δ_f_H_m_^0K^(g) [N] = 470.82 kJ mol^−1^, and Δ_f_H_m_^0K^(g) [O] = 246.79 kJ mol^−1^ were taken from the NIST-JANAF tables [57].

For several isodesmic reactions (in the case of (6)), single-point electronic energies were calculated using the DLPNO-CCSD(T) methodology (the “NormalPNO” truncation thresholds were set) [55] along with the aug-cc-pVQZ basis set [58]. For the sake of brevity, it is denoted henceforth as aVQZ. The RIJK density fitting (DF) approximation [59] was used to accelerate the convergence of the SCF components of DLPNO-CCSD(T) energy. The corresponding auxiliary basis sets (aug-cc-pVQZ/JK and aug-cc-pVQZ/C in the ORCA nomenclature) [49] were used in the DF calculations of the SCF and correlation energies.

The sublimation/evaporation enthalpy was calculated using the refined Trouton-type equation suggested previously [32]. The impact and friction sensitivities for solid samples were obtained according to STANAG standards; the details can be found elsewhere [32]. For liquids, the experimental assembly with free volume was used in line with UN Recommendations on the Transport of Dangerous Goods [60]. The detonation parameters were calculated using the default set of empirical equations implemented in the web application PILEM [61].

## 4. Conclusions

We reported comprehensive syntheses of a bunch of useful high-energetic azidonitrate derivatives (viz., ANDP, SMX, AMDNNM, NIBTN, NPN, 2-nitro-1,3-dinitro-oxypropane). The protocols used are very promising from safety, convenience, and efficiency points of view. Nitroisobutylglycerol was chosen as a common starting reagent for all the compounds, and the procedures were improved to obtain optimal yields and better working conditions. Moreover, a comparative systematic study of their physiochemical and energetic properties was performed. All missing parameters for SMX, NPN, ANDP, AMDNNM, NIBTN, and 2-nitro-1,3-dinitrooxypropane were carefully collected and compared with each other. All compounds were recognized as promising high-energetic fillers.

## Figures and Tables

**Table 1:** Physical and energetic properties of nitroisobutylglycerol derivatives **2**–**7** studied in the present work.

Structure						
	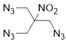	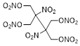	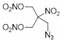	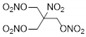	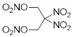	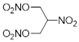
	ANDP **7**	SMX **2**	AMDNNM **6**	NIBTN **3**	NPN **4**	2-Nitro-1,3-dinitrooxypropane **5**
Physicochemical properties						
Formula	C_4_H_6_N_10_O_2_	C_6_H_8_N_6_O_16_	C_4_H_6_N_6_O_8_	C_4_H_6_N_4_O_11_	C_3_H_4_N_4_O_10_	C_3_H_5_N_3_O_8_
M [g mol^−1^] ^[a]^	226.12	420	266.09	286.07	256.1	211.05
N [%] ^[b]^	61.95	20.00	31.58	19.59	21.88	19.91
Ω [%] ^[c]^	−63.7	0	−18.0	0	−12.5	−3.8
*ρ*, g cm^−3 [d]^	1.35 [25] (1.367) [26]	1.973 [43] (1.917) [17,44]	1.55 [26]	1.617 [44]	1.66 [3]	1.760 [24]
$\Delta_{f}H_{m}^{0}$ [kJ mol^−1^] ^[e]^	816	−542 [32]	5	−402	−280	−348
T_m_ [°C] ^[f]^ or T_g_ [°C] ^[g]^	12 (m)	86 (m) [32]	−67.4 (g)	−58.1 (g)	−81.2 (g)	68 (m) [24]
T_dec_. [°C] ^[h]^	179	168 [32]	174	172	167	143
IS [J] ^[i]^	0.3	3 [32]	0.3	0.4	0.4	4
FS [N] ^[j]^	-	50 [32]	-	-	-	120
Detonation parameters						
*D* [m s^−1^] ^[k]^	7.2	9.2	7.8	8.1	7.8	8.4
*P* [GPa] ^[l]^ *C-J*	20	37	25	26	24	29
Heat of explosion, J/g ^[m]^	4657	6260	5460	6160	5310	5840
H ^[n]^	0.74	1.03	0.84	0.89	0.86	0.93

[a] Molar mass; [b] Nitrogen content; [c] Oxygen balance; [d] Density; [e] Standard state enthalpy of formation calculated as a combination of theoretical gas-phase values and empirically estimated sublimation/vaporization enthalpies, more details are given in the Supporting Information; [f] Melting point from DSC (5 K min^−1^, onset); [g] Glass transition temperature; [h] Decomposition temperature from DSC (5 K min^−1^, onset); [i] Impact sensitivity, [j] Friction sensitivity; [k] Detonation velocity; [l] Detonation pressure; [m] Heat of explosion; [n] Relative metal acceleration ability with respect to that for HMX.

## Data Availability

The data presented in this study are available in Supplementary Material here.

## Supplementary Materials

The following supporting information can be downloaded at: https://www.mdpi.com/article/10.3390/ijms24108541/s1.
