# Supplementary material for: Synthesis, Characterization, and Properties of High-Energy Fillers Derived from Nitroisobutylglycerol
Source: Int J Mol Sci. 2023 May 10;24(10):8541. doi: 10.3390/ijms24108541 (PMC10218491; doi:10.3390/ijms24108541)
Supplement: Supplementary file 1 [file ijms-24-08541-s001.zip › ijms-2354472-supplementary.pdf]

Synthesis, Characterization and Properties of High-Energy Compounds from Nitroisobutylglycerol

Supporting information

Sergey A. Rzhevskiy [1], Lidiya I. Minaeva [1], Maxim A. Topchiy [1], Igor N. Melnikov [2], Vitaly G. Kiselev [3], Alla N. Pivkina [2], Igor V. Fomenkov [4] and Andrey F. Asachenko [1,*]

[1] A.V. Topchiev Institute of Petrochemical Synthesis, Russian Academy of Sciences, Leninsky Prospect 29, 119991, Moscow
[2] Semenov Federal Research Center for Chemical Physics RAS, 4 Kosygina Str., 119991 Moscow, Russia
[3] Institute of Chemical Kinetics and Combustion SB RAS, 3 Institutskaya Str., 630090 Novosibirsk, Russia
[4] Zelinsky Institute of Organic Chemistry RAS, 47 Leninsky Ave., 119991 Moscow, Russia
* Correspondence: aasachenko@ips.ac.ru.

Table of content

Thermal behavior upon cooling for liquid samples S2

Thermochemical data S4

NMR spectra S5

Thermal behavior upon cooling for liquid samples

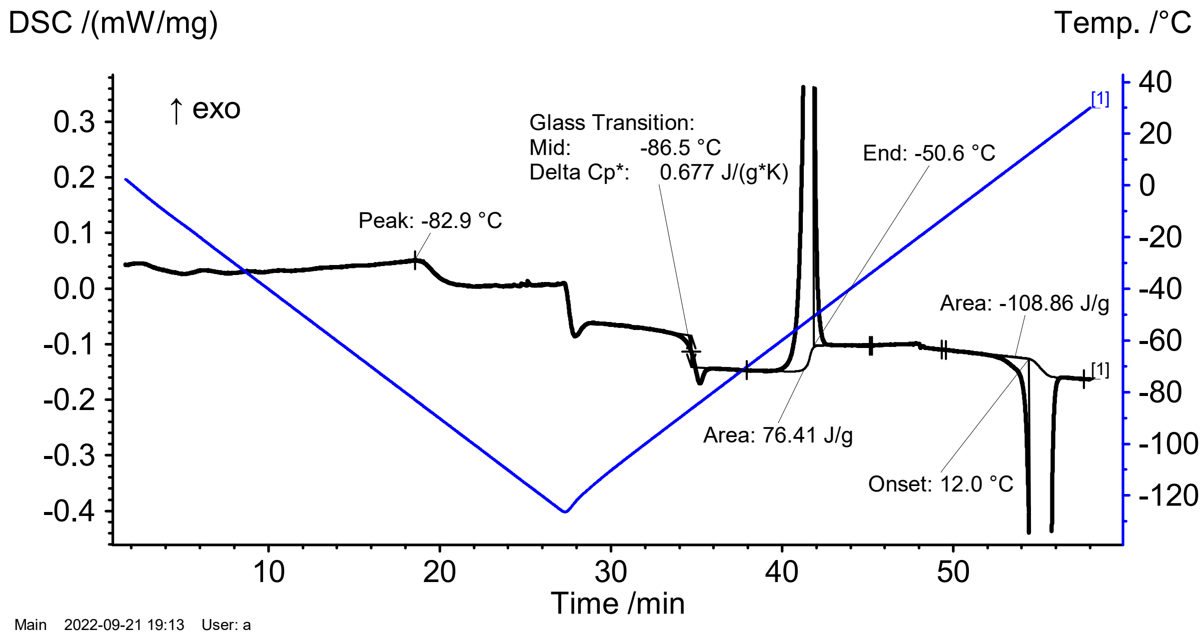

Figure S1. DSC (black curve) and a temperature time dependence (blue line) for the cooling-heating cycle of ANDP (7). The cooling and heating rates were 5 K min^{-1}. From left to the right: a vitrification effect, a DSC trace change due to a temperature program change (an experimental artefact), glass transition, cold crystallization, and melting (m.p. is 12°C).

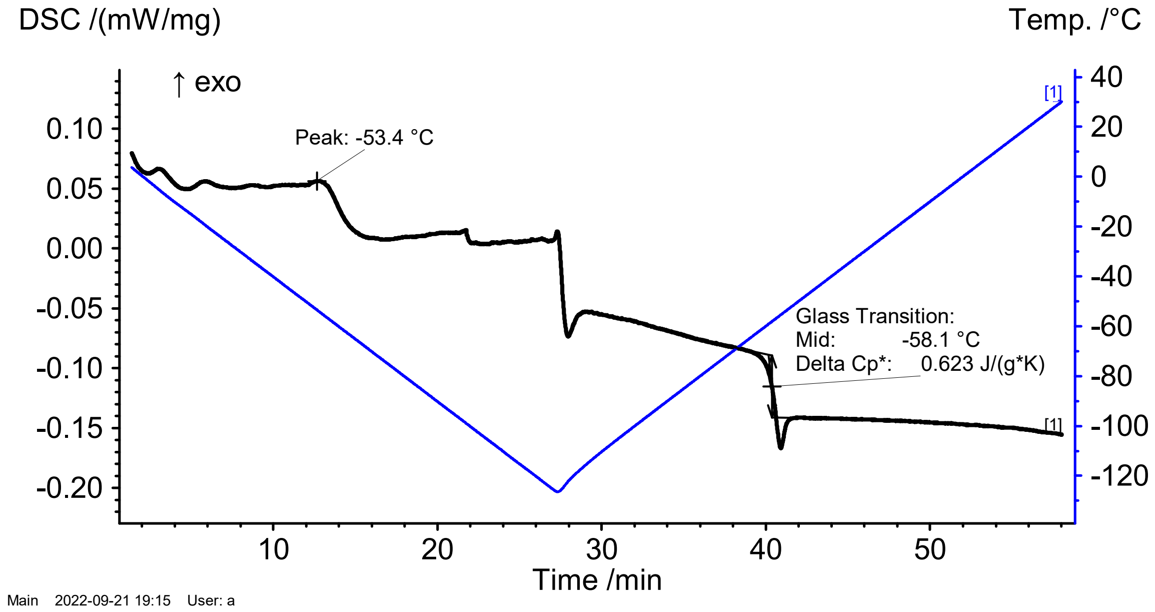

Figure S2. DSC (black curve) and a temperature time dependence (blue line) for the cooling-heating cycle of NIBTN (3). The cooling and heating rates were 5 K min^{-1}. From left to the right: a vitrification effect, DSC change due to a temperature program change (an experimental artefact), glass transition.

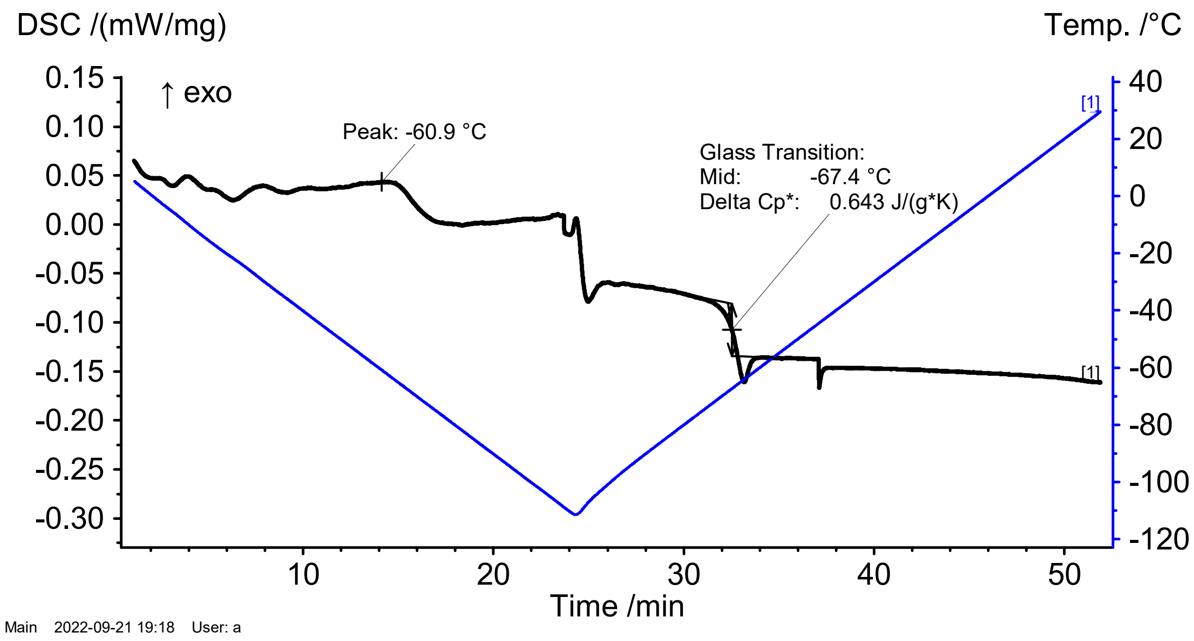

Figure S3. DSC (black curve) and a temperature time dependence (blue line) for the cooling-heating cycle of AMDNNM (6). The cooling and heating rates were 5 K min^{-1}. From left to the right: a vitrification effect, DSC change due to a temperature program change (an experimental artefact), glass transition.

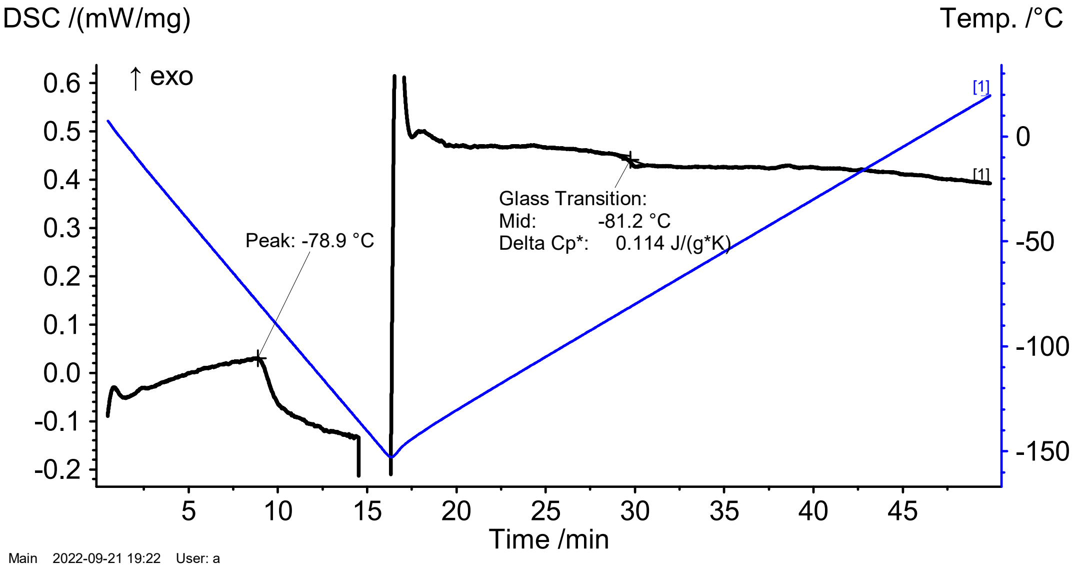

Figure S4. DSC (black curve) and a temperature time dependence (blue line) for the cooling-heating cycle of NPN (4). The cooling and heating rates were 5 K min^{-1}. From left to the right: vitrification effect, DSC change due to temperature program change (an experimental artefact), glass transition.

Thermochemical data

The sublimation enthalpies of the species studied were calculated using the modified Trouton rule proposed recently [18]. In the case of ANDP (7) and 2-Nitro-1,3-dinitro-oxypropane **5** we used the melting points from our DSC experiments, for all other compounds – those estimated from the glass transition temperature values. In order to obtain the evaporation enthalpy for a particular liquid at room temperature, we used either the DSC melting enthalpy (in the case of ANDP) or the average value of the literature values -110 J/g for ANDP, SMX, PETN, ETN from this study and Ref 18 from the paper. Standard state enthalpies of formation listed in Table S1 were calculated as a combination of theoretical gas-phase values and empirically estimated sublimation/vaporization enthalpies. In the particular case of (6) the following isodesmic reaction was used:

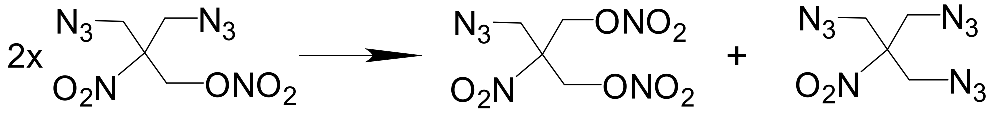

6a **6** **7** (R1)

Combining the DLPNO-CCSD(T)/aVQZ enthalpy of this reaction 1.4 kJ/mol with the $\Delta_f H_m^0(gas)$ values of 510.3 and 922.0 kJ/mol obtained for (6a) and (7), respectively, using the atomization energies calculated using the W1-F12 multi-level procedure, finally we obtained the resulting $\Delta_f H_m^0(gas)$ of (6) to be 100 kJ/mol.

Table S1. The theoretically calculated gas-phase formation enthalpies $\Delta_f H_m^0(gas)$, empirically estimated sublimation/vaporization enthalpies $\Delta_{sub} H_m^0$ and resulting standard-state formation enthalpies $\Delta_f H_m^0$. All values are in kJ mol[-1].

	Structure					
	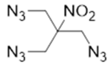	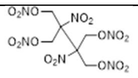	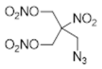	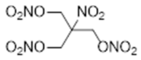	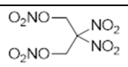	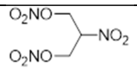
	ANDP (7)	SMX (2)	AMDNNM (6)	NIBTN (3)	NPN (4)	2-Nitro-1,3-dinitro-oxypropane (5)
Formula	$C_4H_6N_{10}O_2$	$C_6H_8N_6O_{16}$	$C_4H_6N_6O_8$	$C_4H_6N_4O_{11}$	$C_3H_4N_4O_{10}$	$C_3H_5N_3O_8$
$\Delta_f H_m^0(gas)$	922 [a]		100 [b]	-308 [a]	-197 [a]	-227 [a]
$\Delta_{sub} H_m^0$	106		95	94	83	121
$\Delta_f H_m^0$ [c]	816	-542 [18]	5	-402	-280	-348

[a] Calculated using the W1-F12 multi-level procedure along with the atomization energy approach. [b] Calculated using the isodesmic reaction (R1). [c] Standard state enthalpy of formation calculated as a combination of theoretical gas-phase values and empirically estimated sublimation/vaporization enthalpies.

NMR spectra

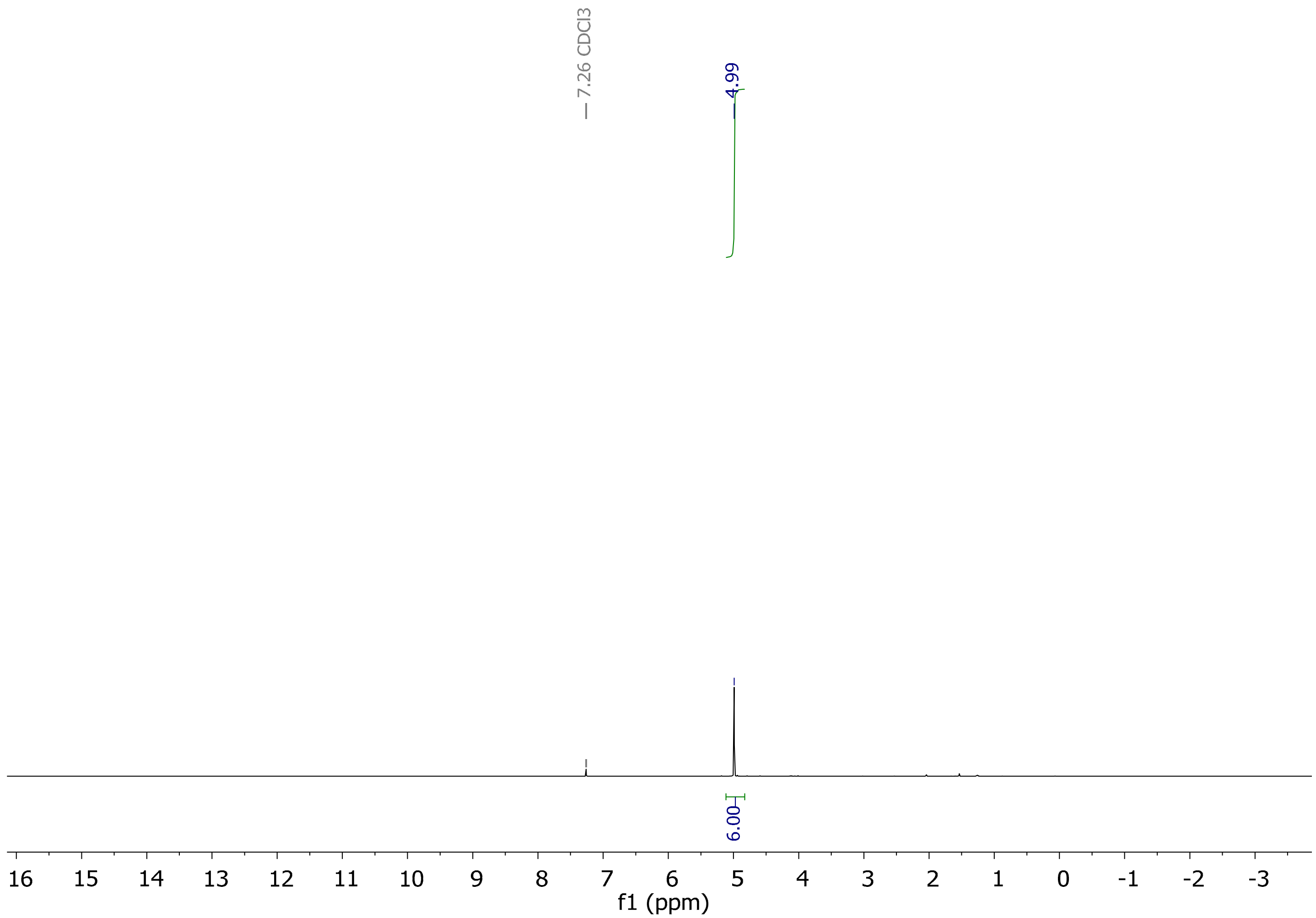

Figure S5. ^{1}H NMR (400 MHz, $CDCl_3$) spectra of trimethylol nitromethane trinitrate NIBTN (3).

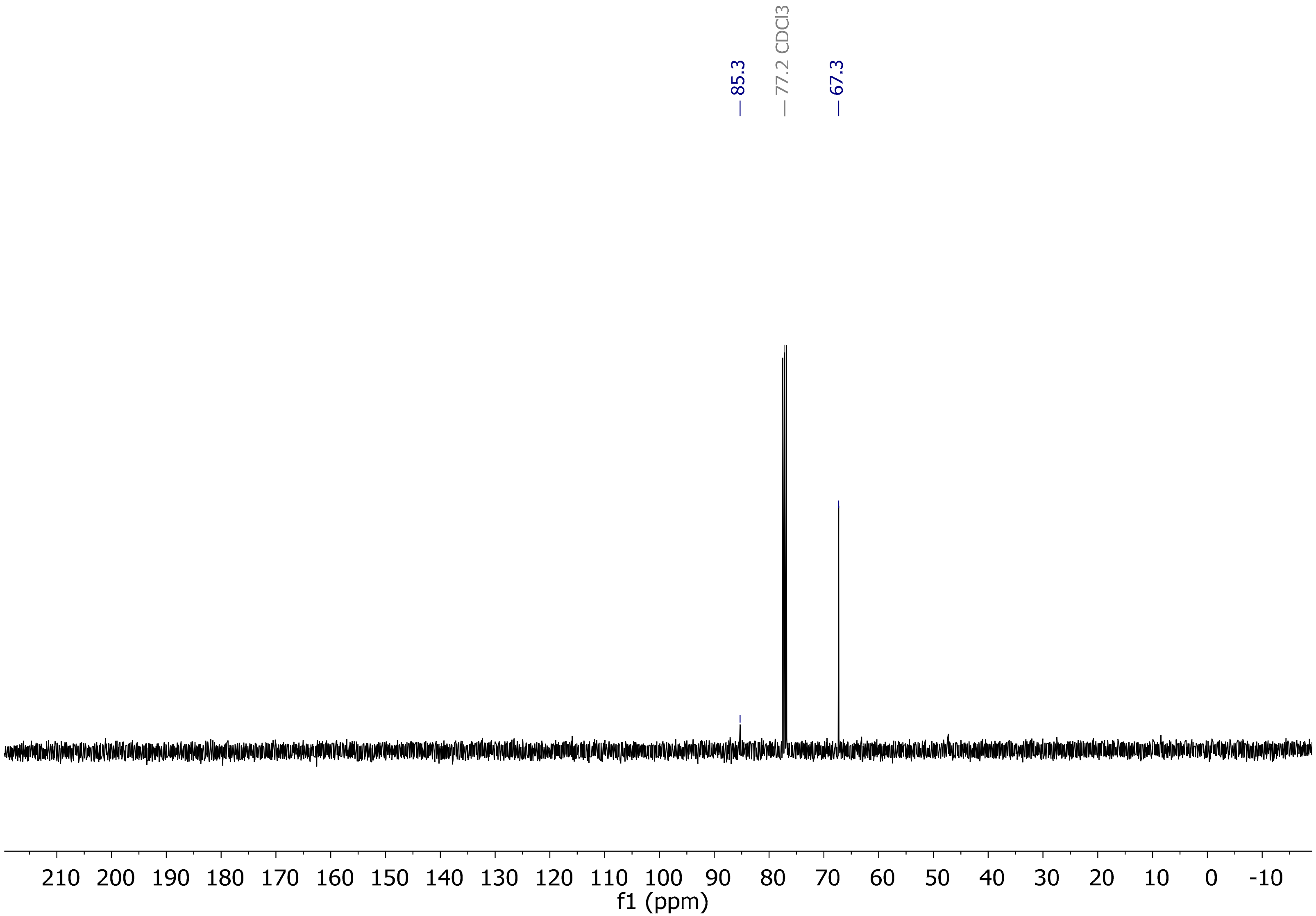

Figure S6. $^{13}C\{^{1}H\}$ NMR (101 MHz, $CDCl_3$) spectra of trimethylol nitromethane trinitrate NIBTN (3).

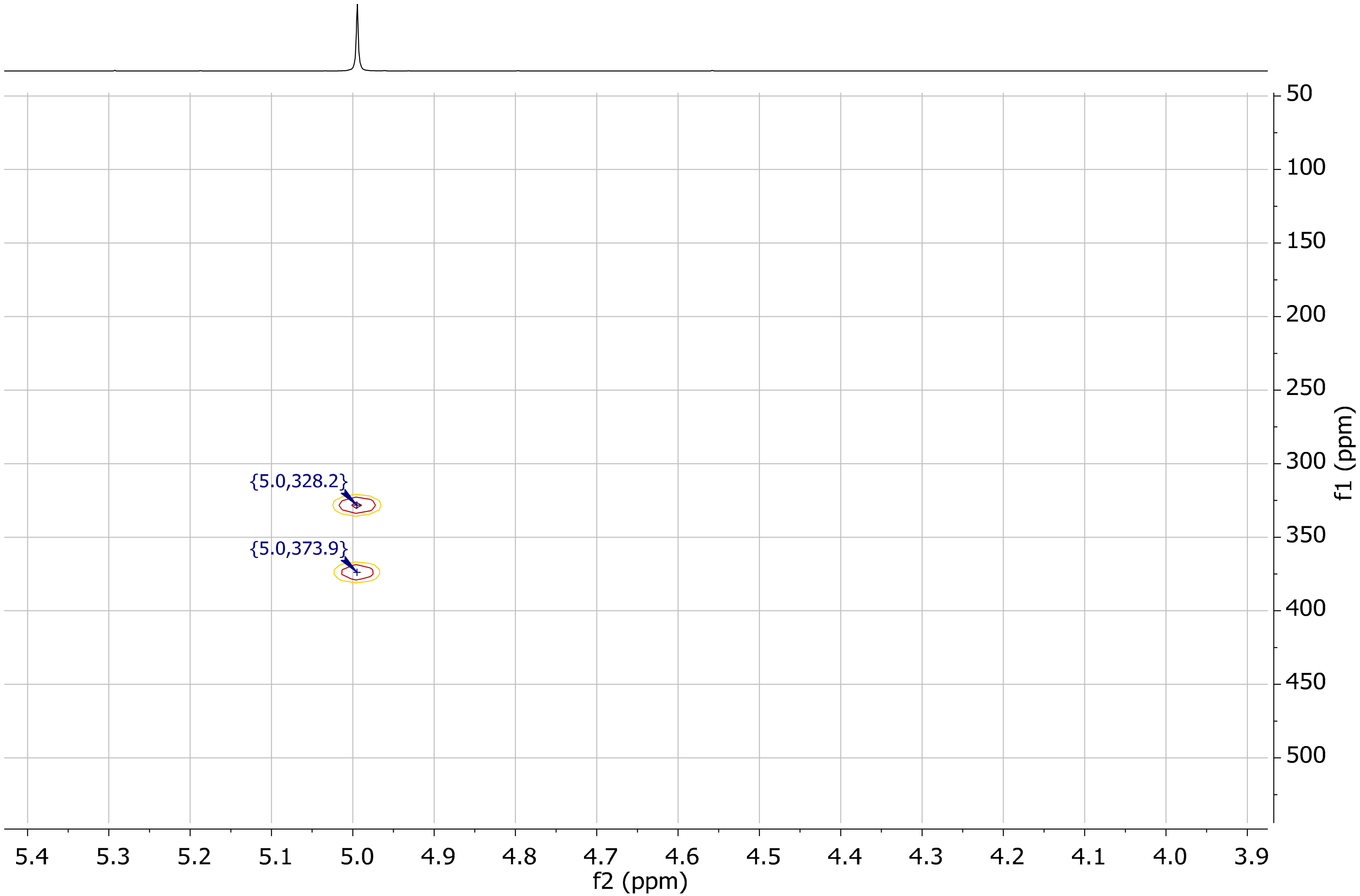

Figure S7. HMBC ^{1}H-^{15}N (400 MHz, 40.55 MHz, $CDCl_3$) spectra of NIBTN (3).

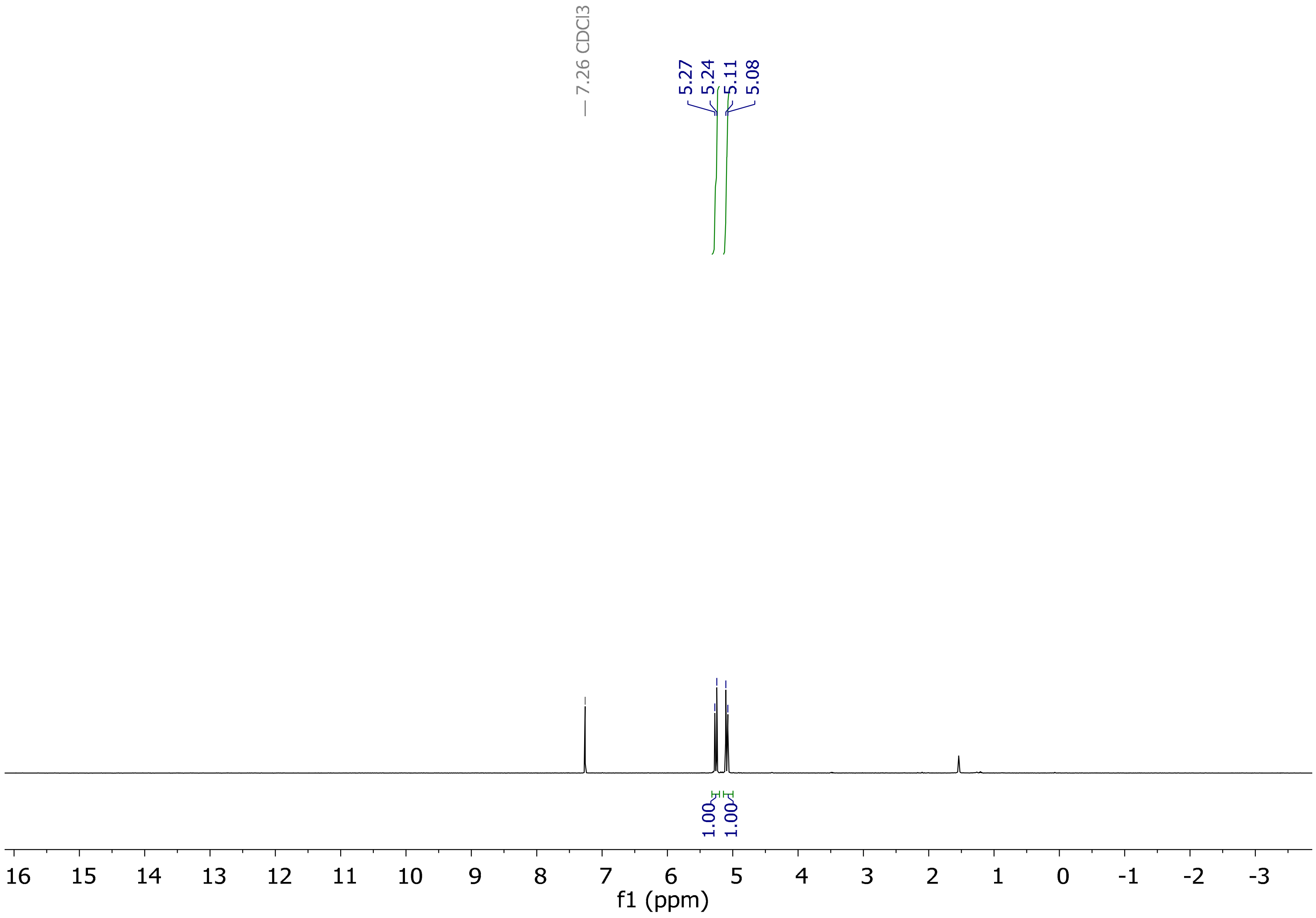

Figure S8. ^{1}H NMR (400 MHz, $CDCl_3$) spectra of SMX (2).

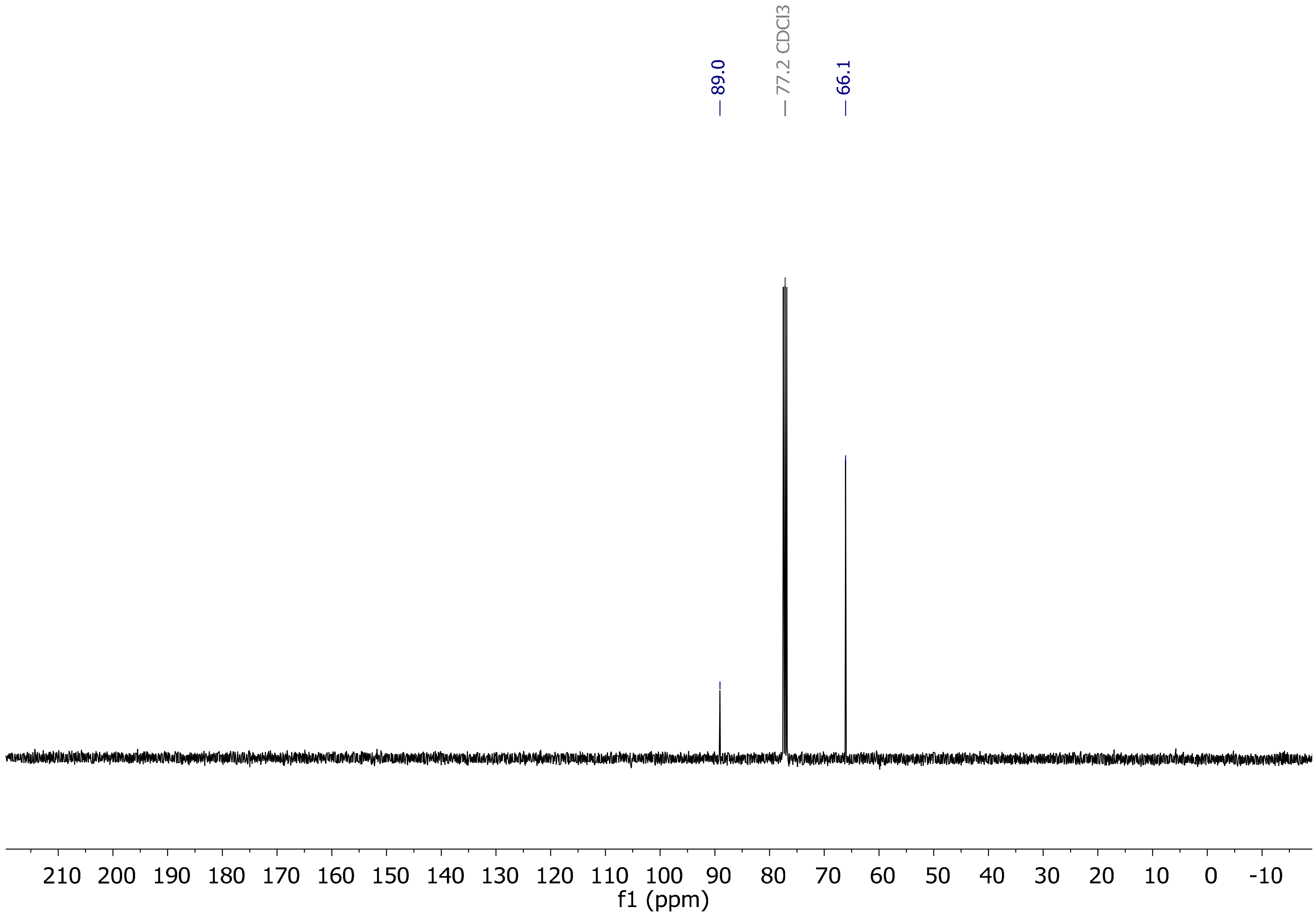

Figure S9. $^{13}C\{^{1}H\}$ NMR (101 MHz, $CDCl_3$) spectra of SMX (2).

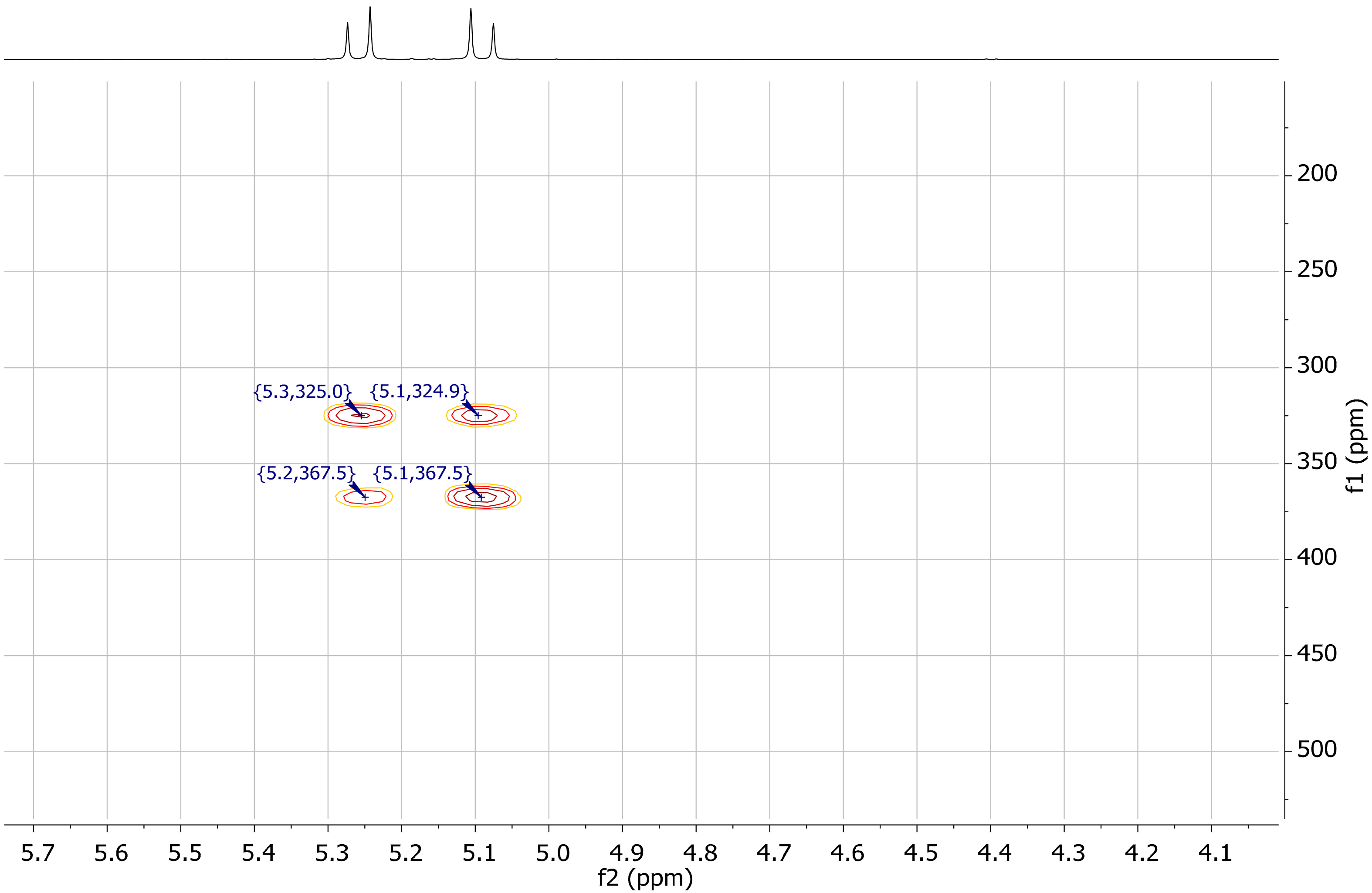

Figure S10. HMBC ^{1}H-^{15}N (400 MHz, 40.55 MHz, $CDCl_3$) spectra of SMX (2).

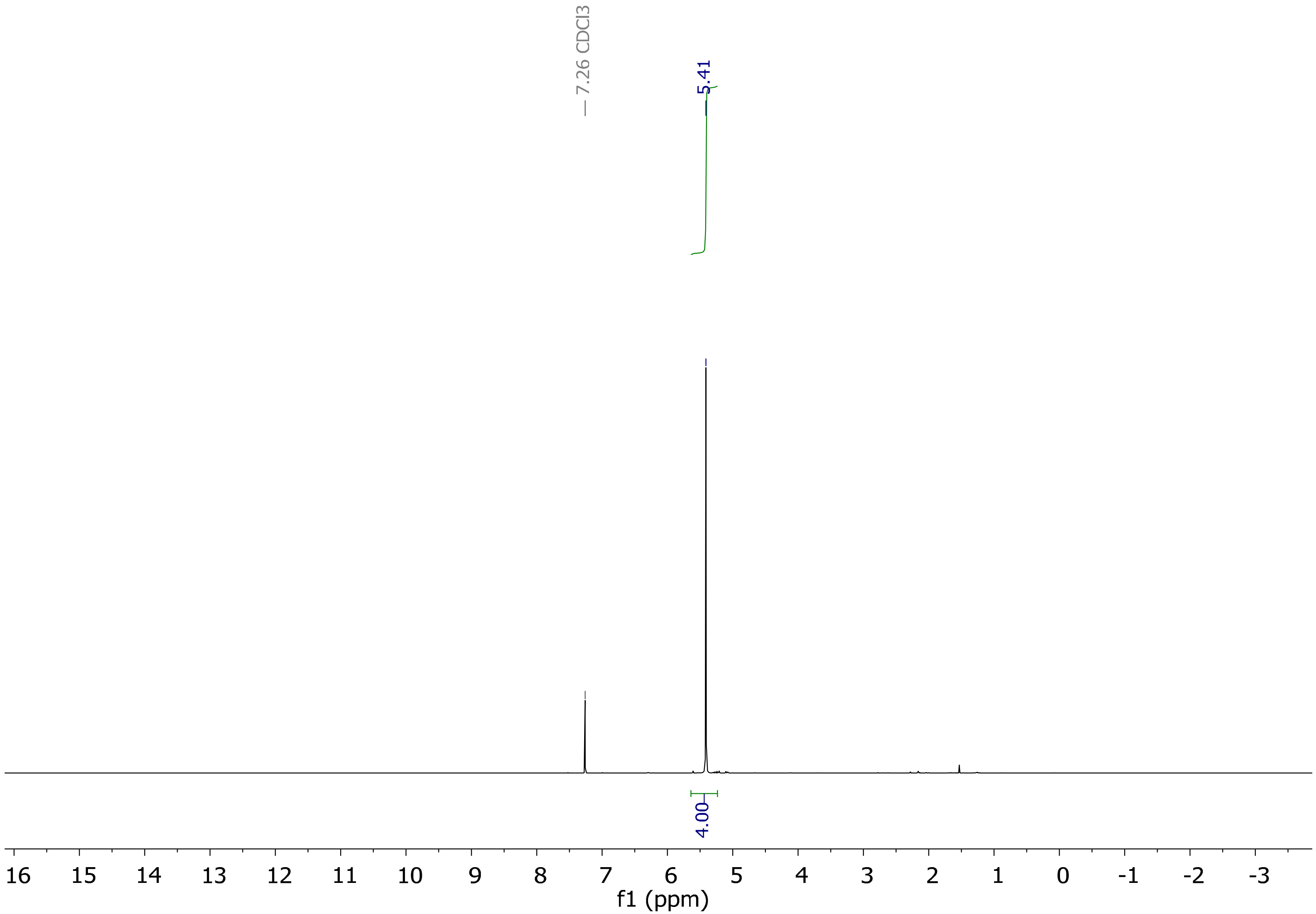

Figure S11. ^{1}H NMR (400 MHz, $CDCl_3$) spectra of 2,2-dinitropropane-1,3-diyl dinitrate (NPN) **4.**

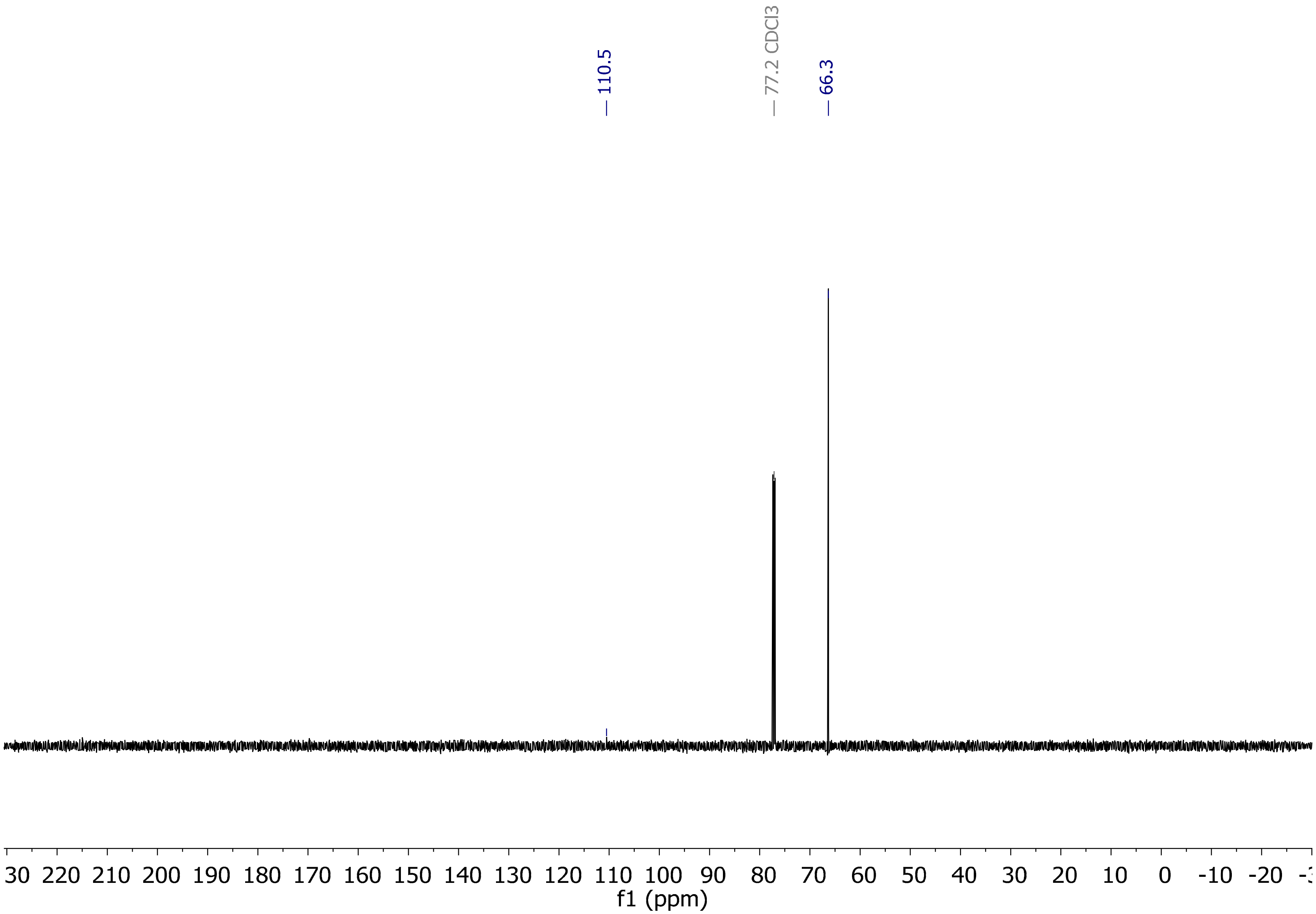

Figure S12. $^{13}C\{^1H\}$ NMR (101 MHz, $CDCl_3$) spectra of 2,2-dinitropropane-1,3-diyl dinitrate (NPN) **4.**

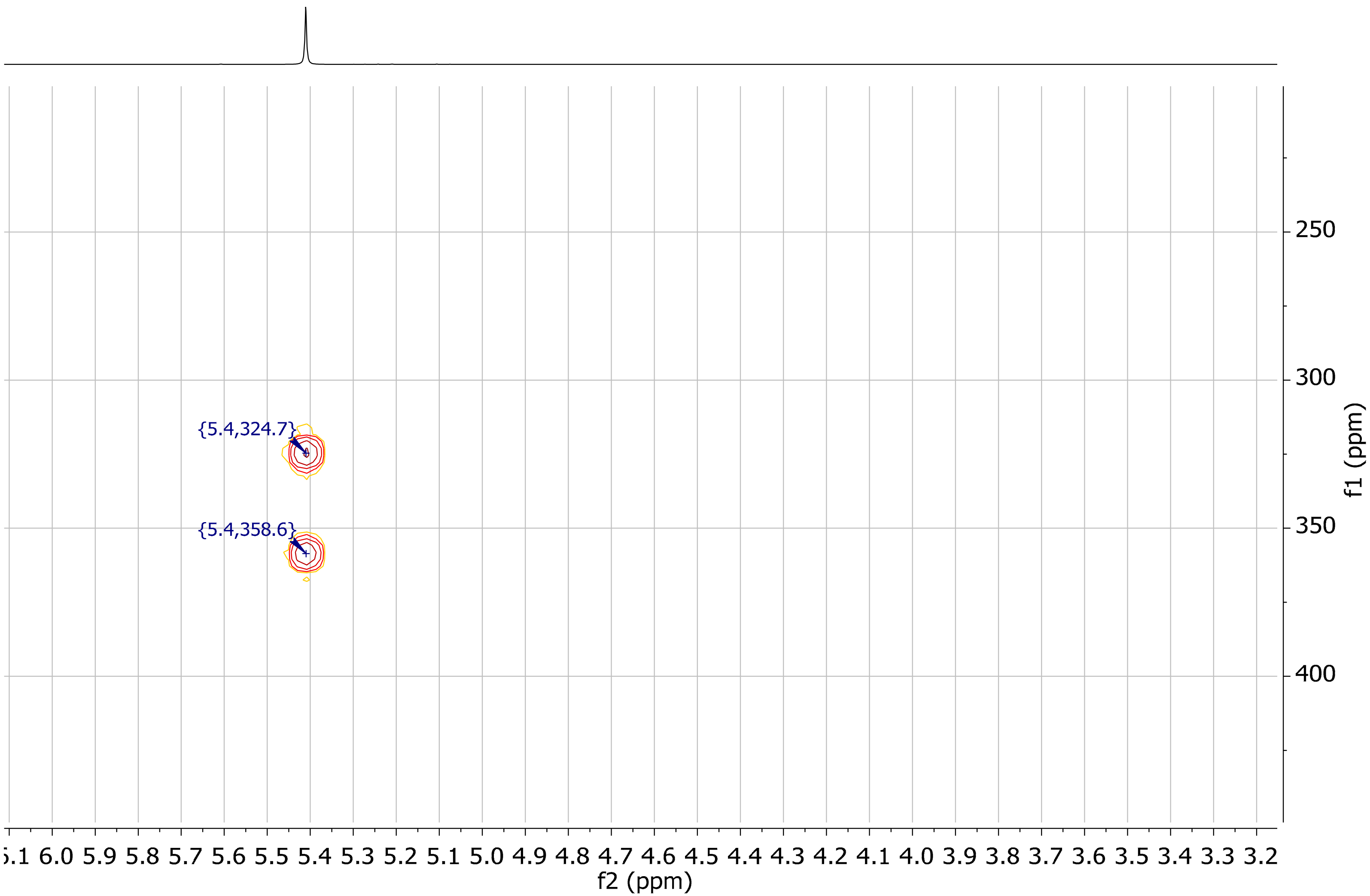

Figure S13. HMBC ^{1}H-^{15}N (400 MHz, 40.55 MHz, $CDCl_3$) spectra of 2,2-dinitropropane-1,3-diyl dinitrate (NPN) **4.**

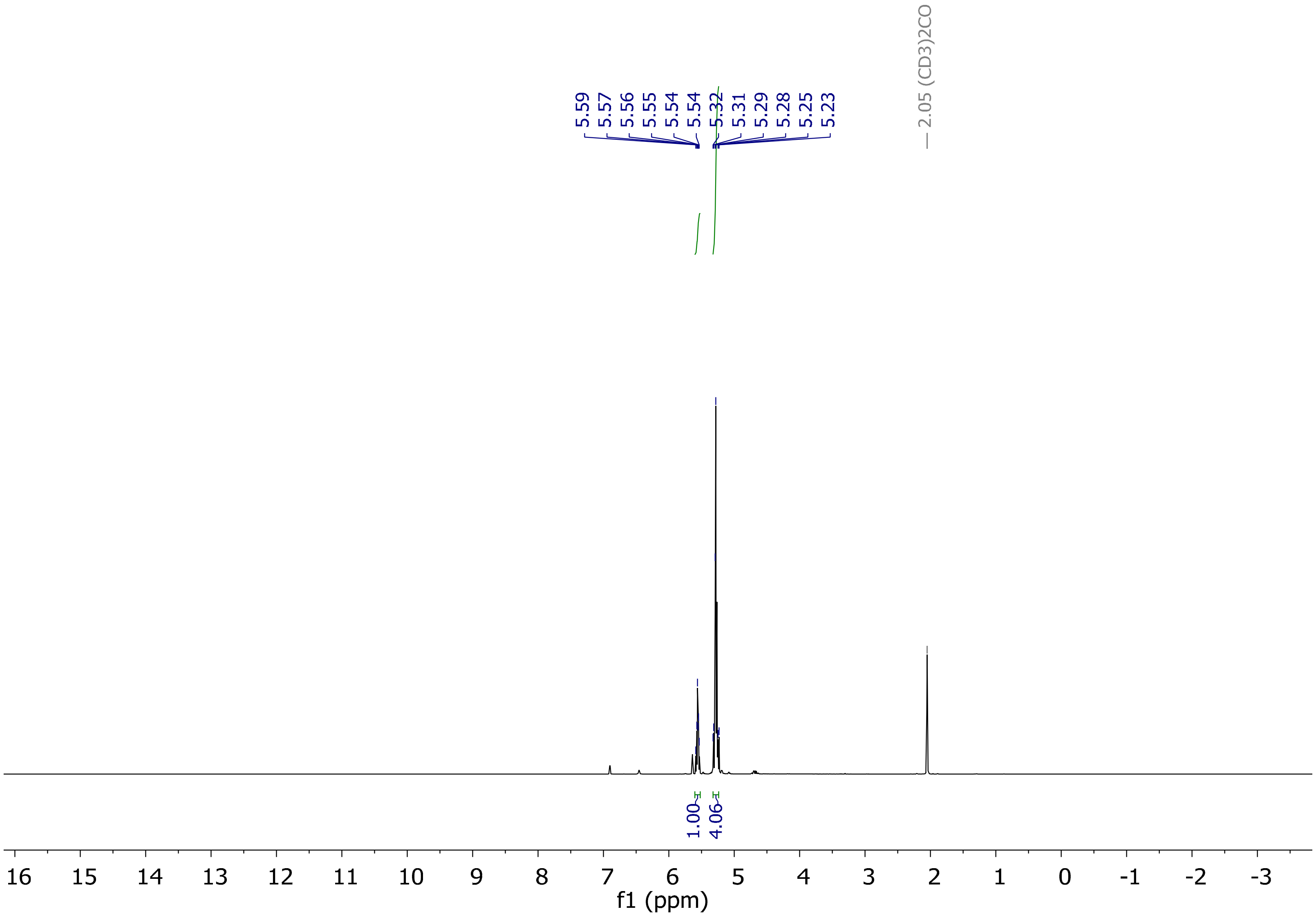

Figure S14. ^{1}H NMR (400 MHz, acetone-d6) spectra of 2-nitro-1,3-dinitrooxypropane (5).

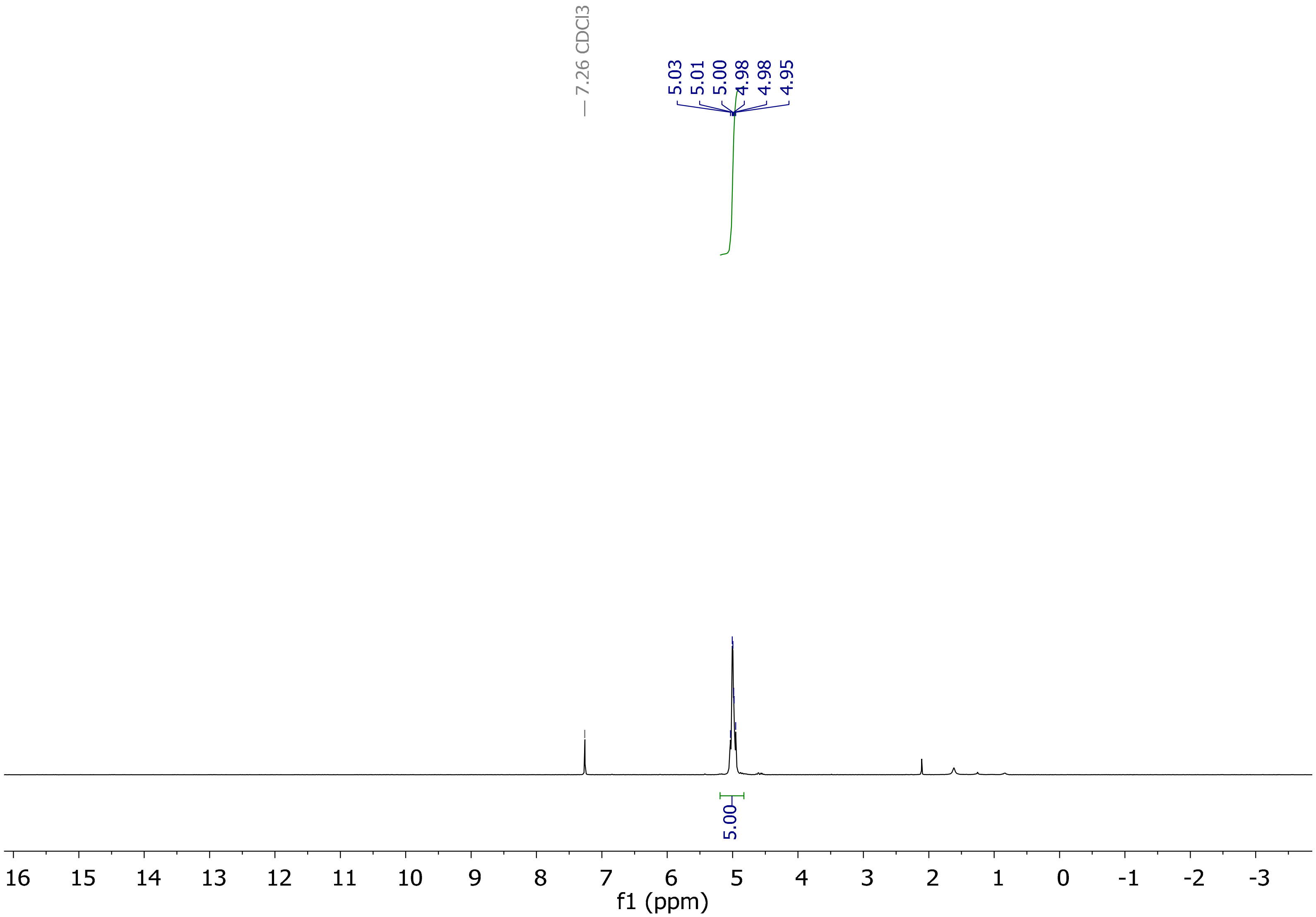

Figure S15. ^{1}H NMR (400 MHz, $CDCl_3$) spectra of 2-nitro-1,3-dinitrooxypropane **5.**

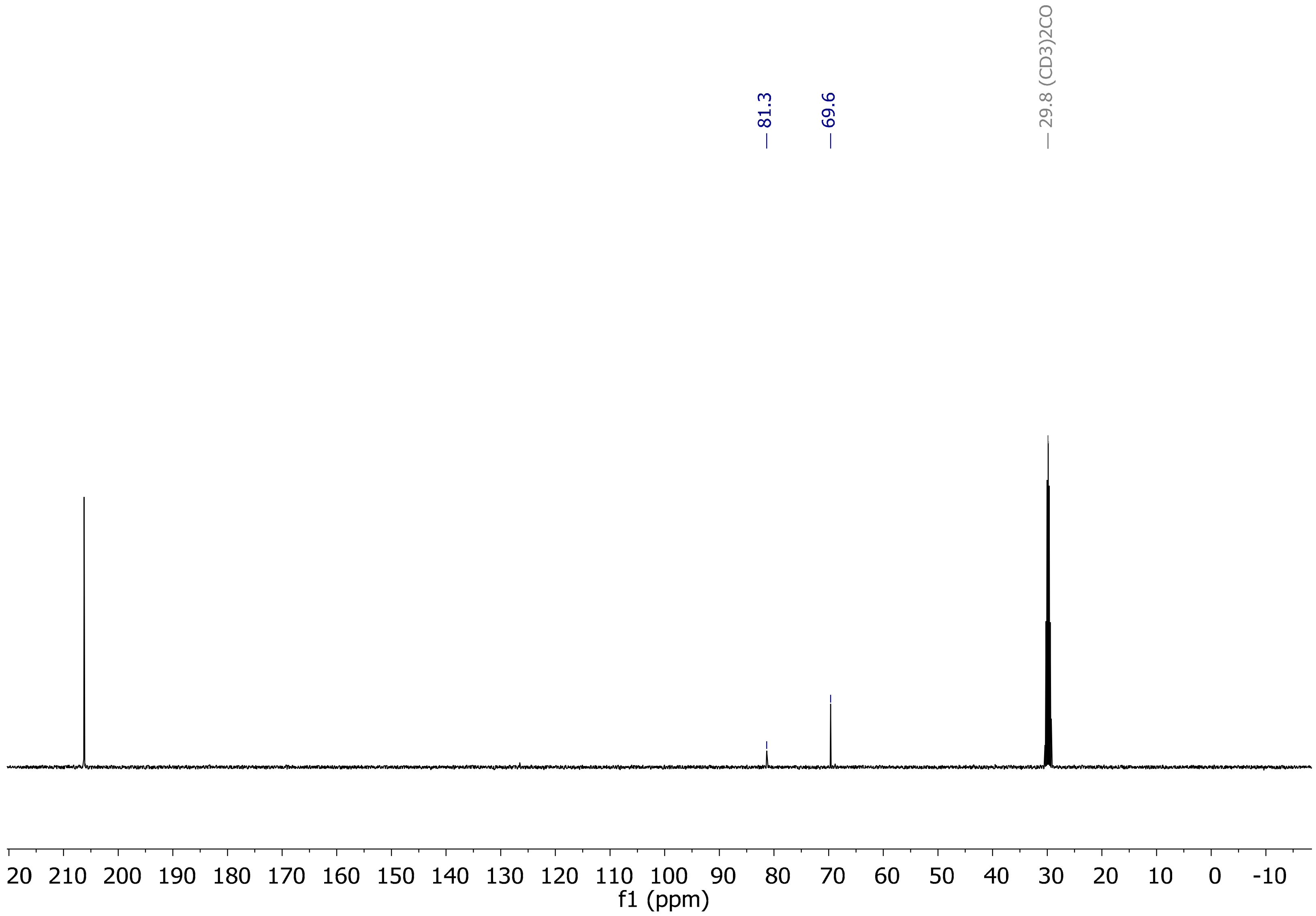

Figure S16. $^{13}C\{^1H\}$ NMR (101 MHz, acetone-d6) spectra of 2-nitro-1,3-dinitrooxypropane (5).

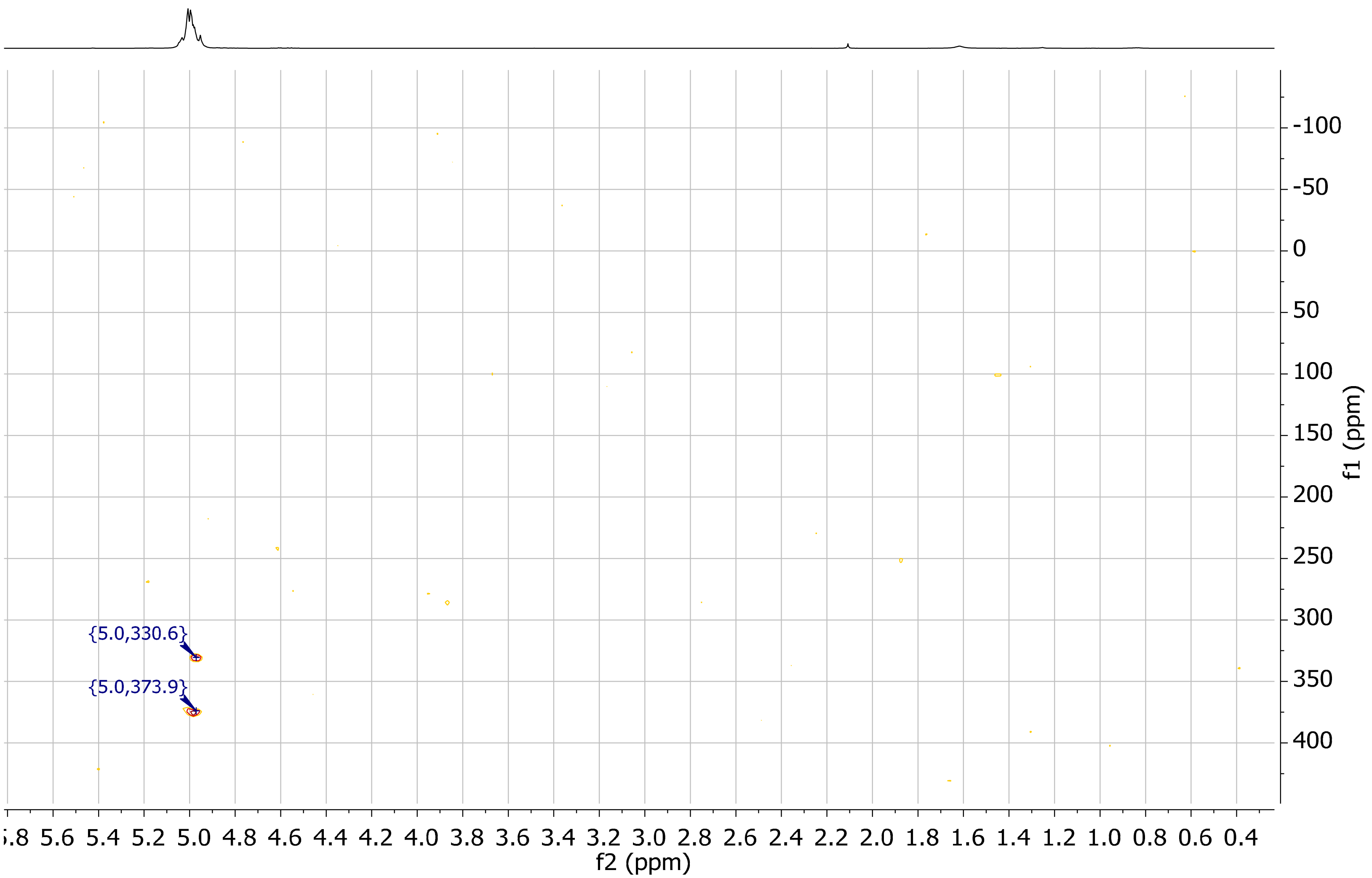

Figure S17. HMBC ^{1}H-^{15}N (400 MHz, 40.55 MHz, $CDCl_3$) spectra of 2-nitro-1,3-dinitrooxypropane (5).

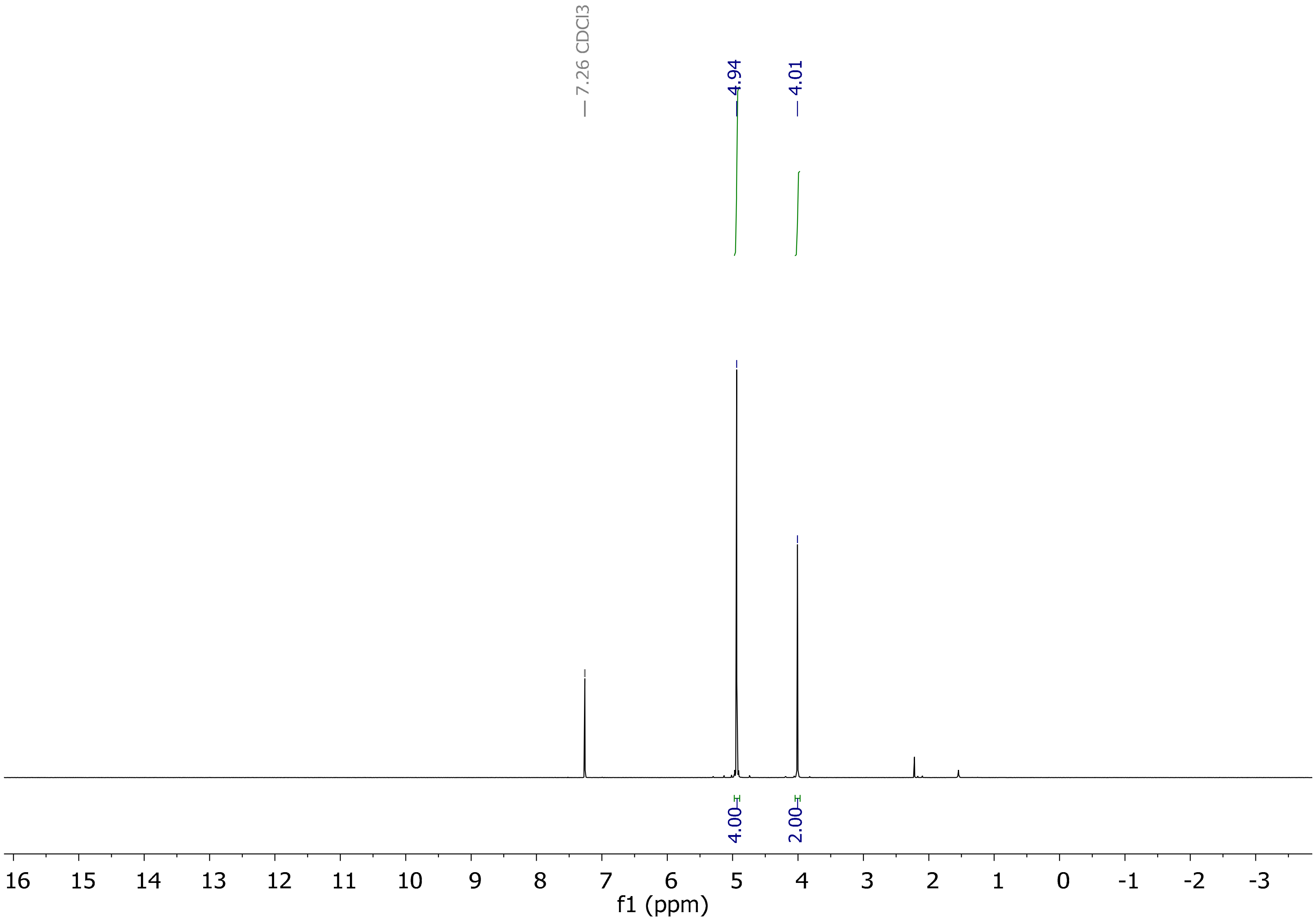

Figure S18. ^{1}H NMR (400 MHz, $CDCl_3$) spectra of AMDNNM (6).

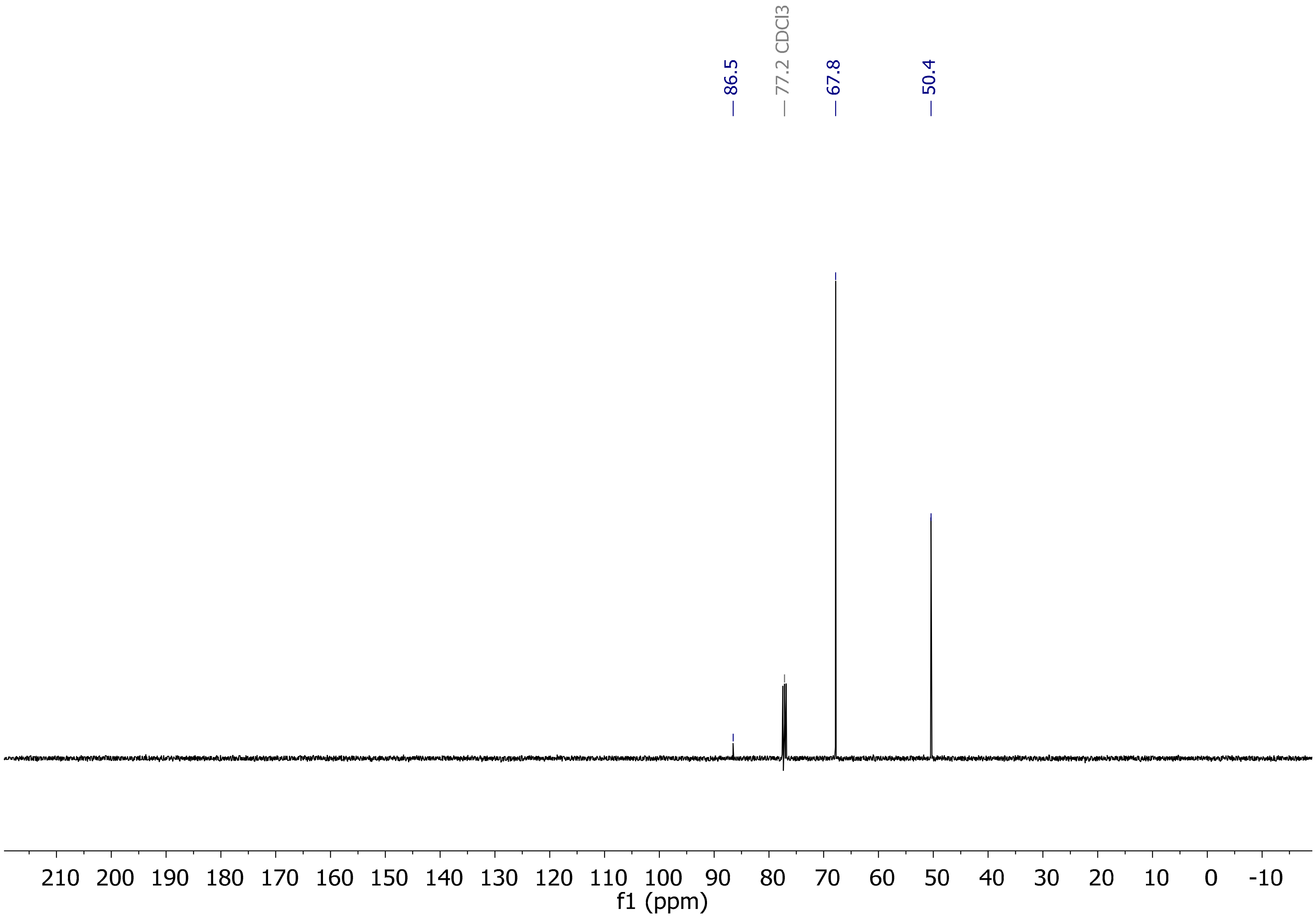

Figure S19. $^{13}C\{^{1}H\}$ NMR (101 MHz, $CDCl_3$) spectra of AMDNNM (6).

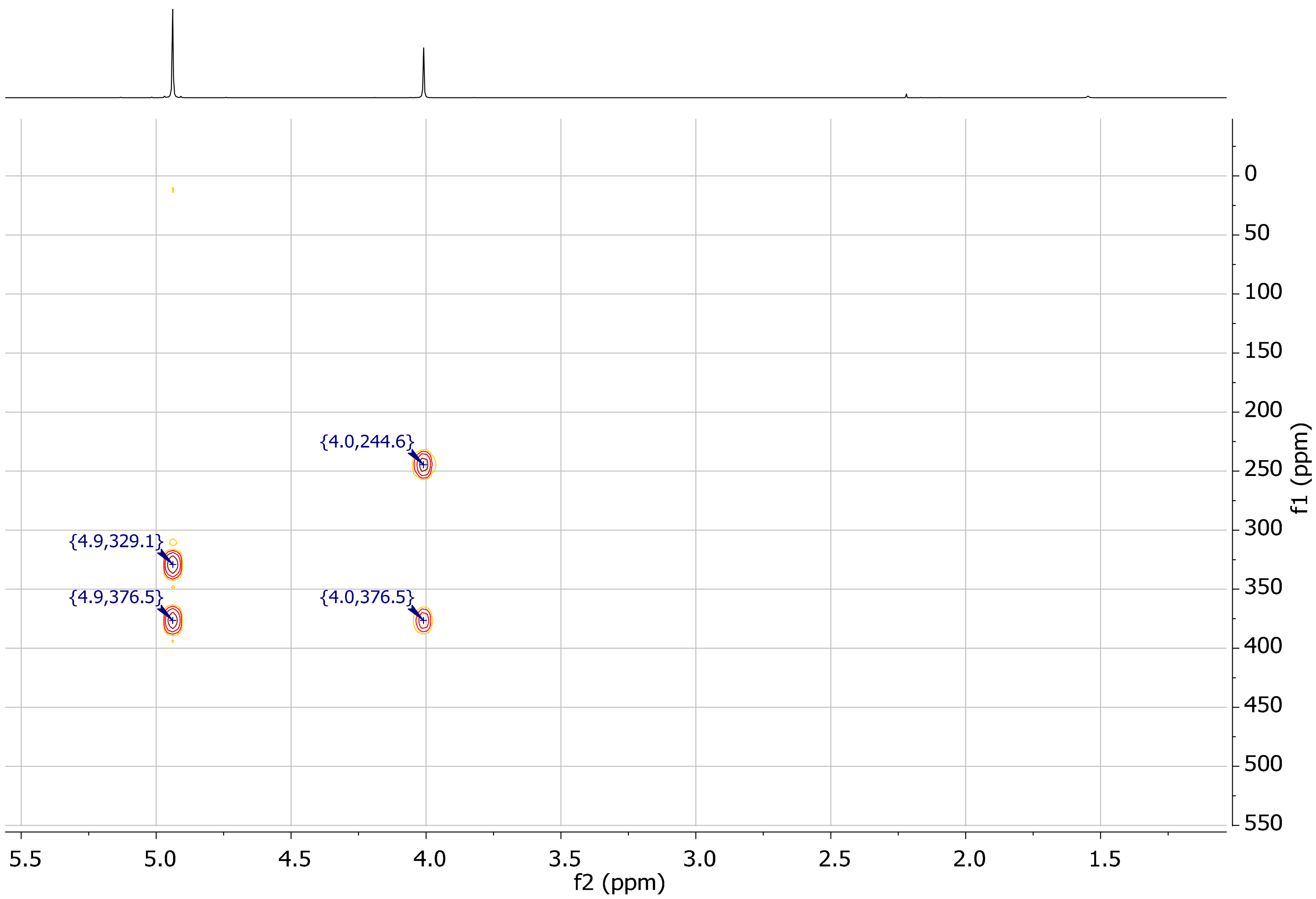

Figure S20. HMBC ^{1}H-^{15}N (400 MHz, 40.55 MHz, $CDCl_3$) spectra of AMDNNM (6).

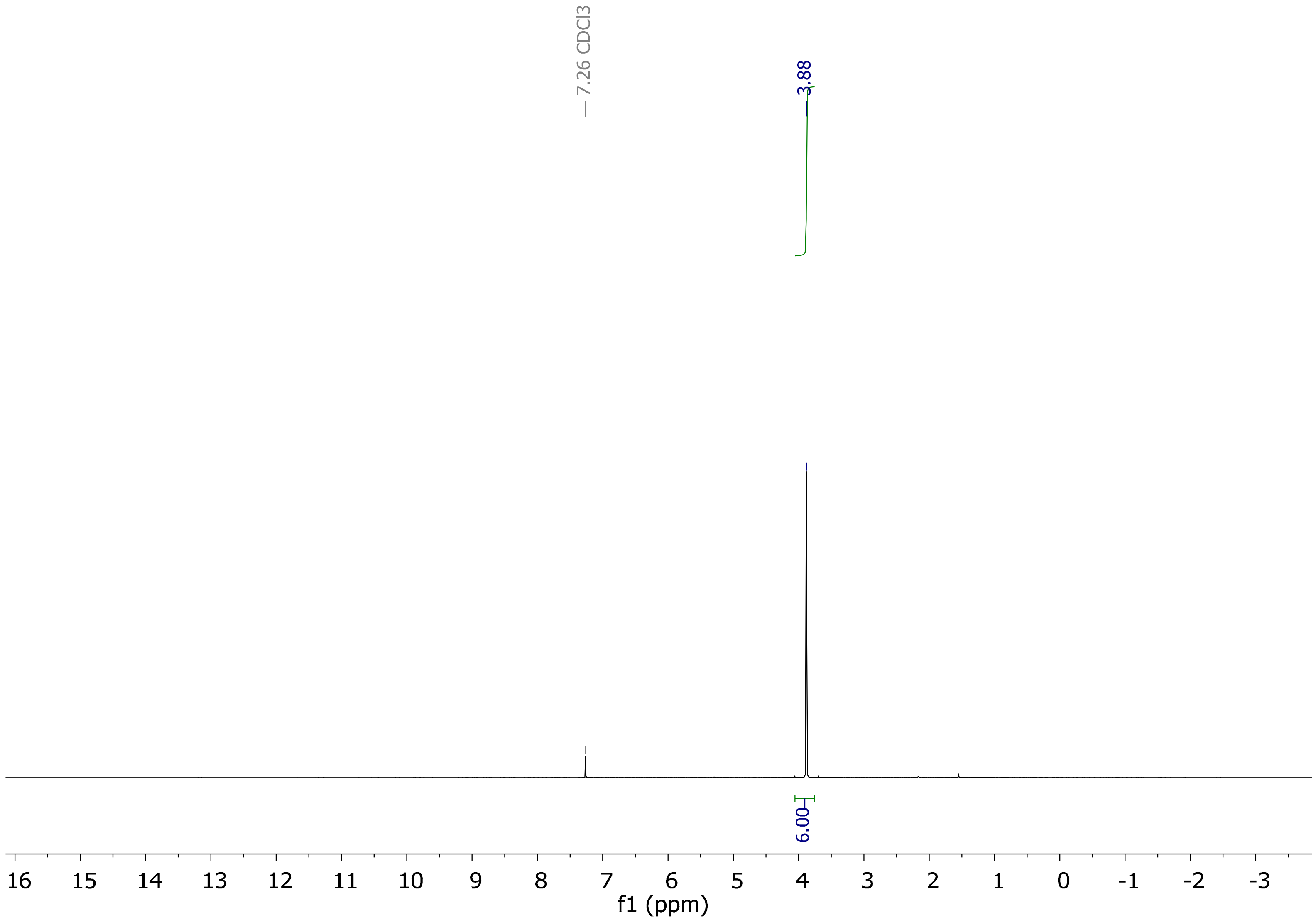

Figure S21. ^{1}H NMR (400 MHz, $CDCl_3$) spectra of 1,3-diazido-2-(azidomethyl)-2-nitropropane (ANDP) **7.**

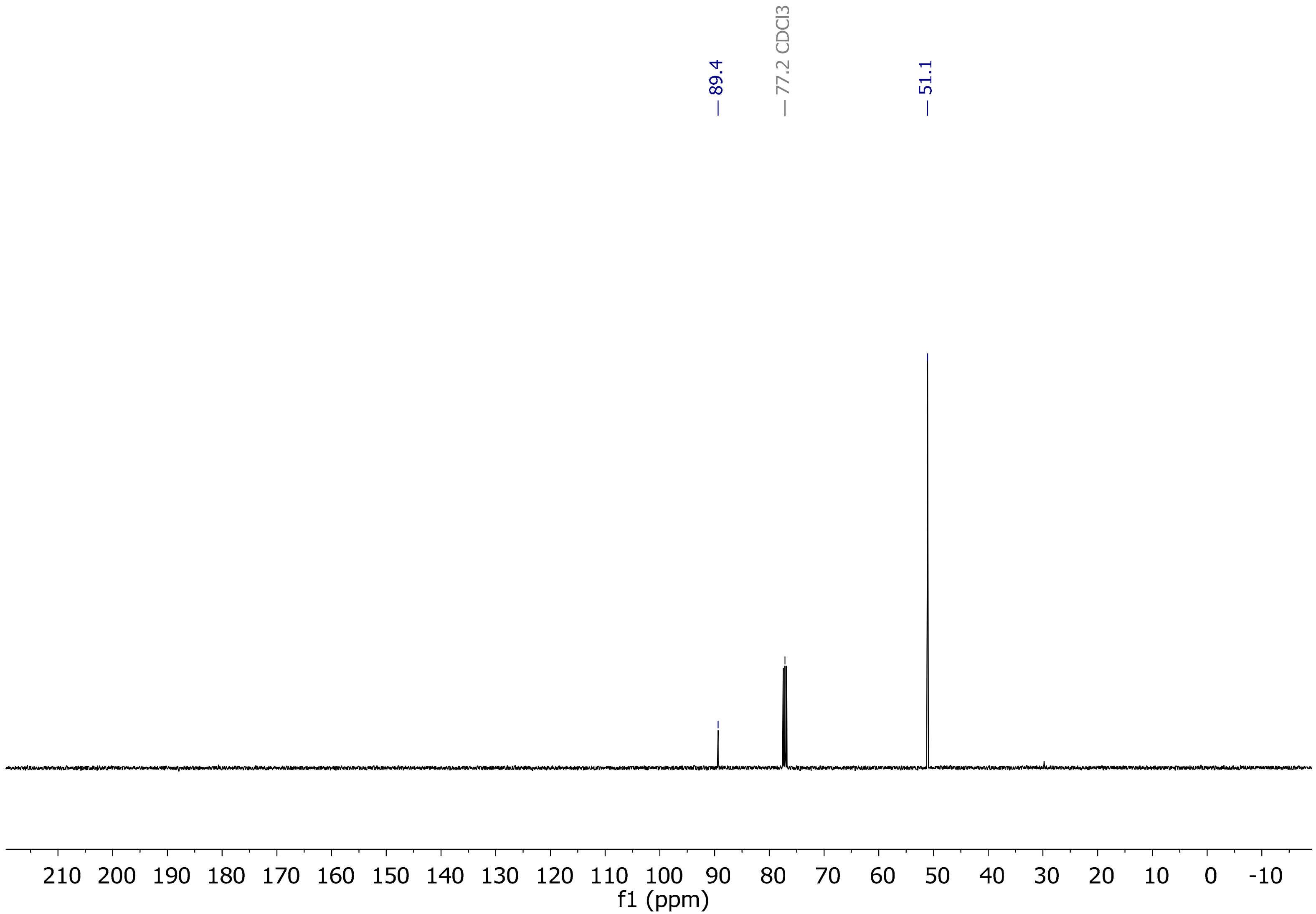

Figure S22. $^{13}C\{^{1}H\}$ NMR (101 MHz, $CDCl_3$) spectra of 1,3-diazido-2-(azidomethyl)-2-nitropropane (ANDP) **7.**

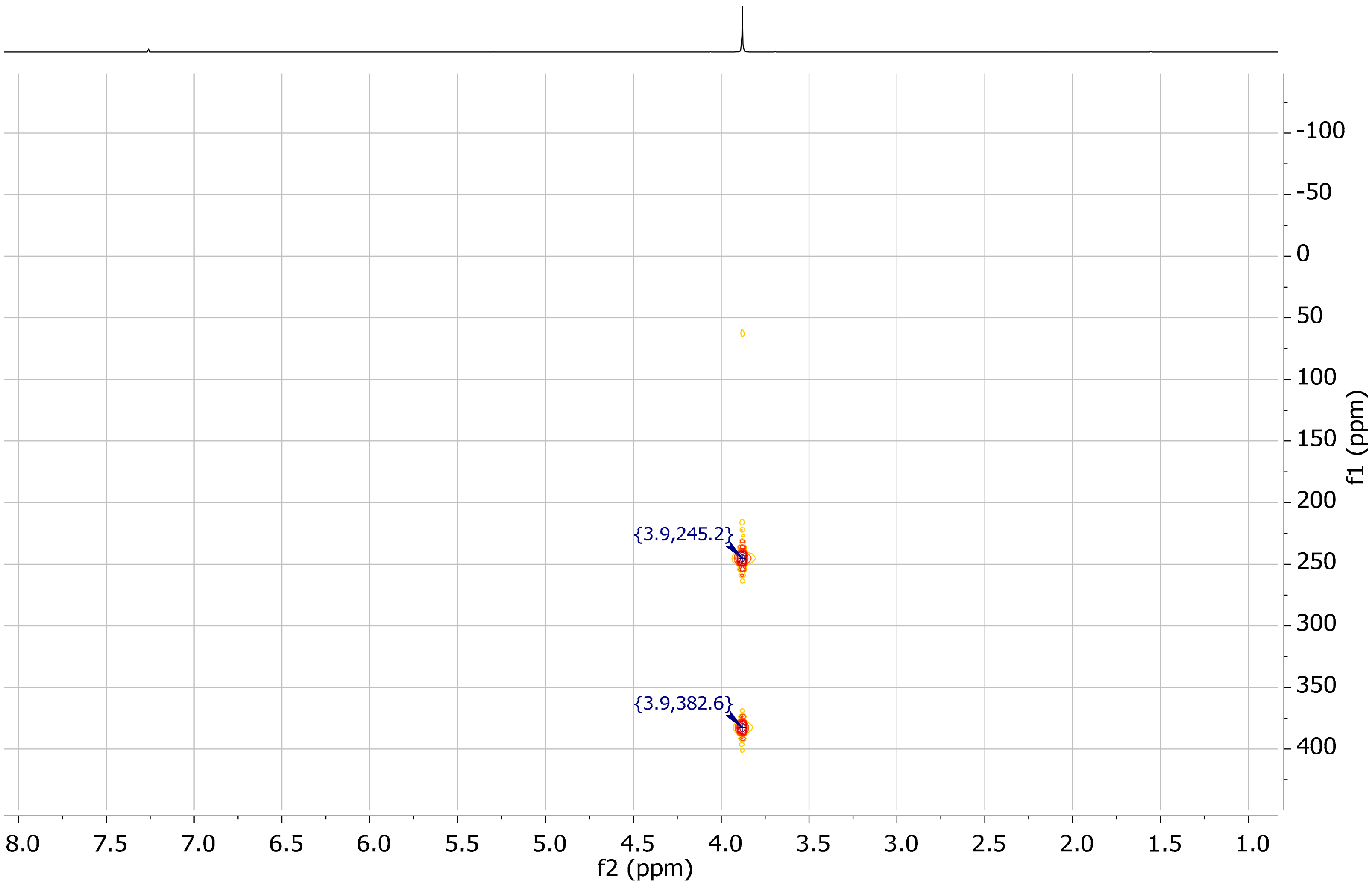

Figure S23. HMBC ^{1}H-^{15}N (400 MHz, 40.55 MHz, $CDCl_3$) spectra of 1,3-diazido-2-(azidomethyl)-2-nitropropane (ANDP) **7**.
